# Recovering scheduling preferences in dynamic departure time models

**DOI:** 10.1140/epjds/s13688-025-00608-z

**Published:** 2026-03-03

**Authors:** Zhenyu Yang, Pietro Giardina, Nikolas Gerolimnis, André de Palma

**Affiliations:** 1 Urban Transport Systems Laboratory (LUTS), École Polytechnique Fedéralé de Lausanne (EPFL), Lausanne, 1015 Switzerland; 2 THEMA, CY Cergy Paris Université, Cergy-Pontoise, 95011 France

**Keywords:** Bottleneck, Scheduling preferences, Traffic flow, Travel demand management, C25, R41, D12

## Abstract

We aim to infer commuters’ scheduling preferences from their observed arrival times, given an exogenous traffic congestion pattern. To do this, we employ a structural model that characterizes how users balance congestion costs against the penalties for arriving early or late relative to an ideal time. In this framework, each commuter selects an arrival time that minimizes her overall trip cost by considering the within-day congestion pattern along with her individual scheduling preference. By incorporating the distribution of these preferences and desired arrival times across the population, we can estimate the likelihood of observing arrivals at specific times. Using synthetic data, we then apply the maximum likelihood estimation (MLE) method to recover the parameters of the joint distribution of scheduling preferences and desired arrival times. Our numerical results demonstrate the effectiveness of the proposed method.

## Introduction

Travel demand management (TDM) schemes play a crucial role in mitigating urban congestion by shaping when, how, and by which mode individuals travel. Urban mobility can be understood as a complex system governed by the interactions of three main actors: travelers, who decide on departure time, route, and mode; governments, which design, regulate, and price infrastructure through policies such as tolls, emissions standards, and access restrictions; and service providers, who adapt transportation supply in response to demand and capacity constraints. The objectives of these actors often diverge, and insufficient coordination both within and across these groups generates systemic inefficiencies ranging from congestion and underutilized capacity to inequitable access (see the discussion on policy analysis in the recent textbook: [1]).

Implementing effective TDM strategies requires a solid understanding of travelers’ behavior and their responsiveness to policy interventions. Because traffic emerges from countless individual decisions made across large and interconnected networks, modelling tools are essential for guiding policymakers. Simple analytical models are valuable for generating key policy insights, while large-scale simulation models are needed to capture the systemic and network-wide dimensions of transportation.

Transportation modelling initially relied on static frameworks, which assume that congestion levels remain constant over time, typically by treating peak and off-peak periods as two independent equilibria. Despite this restrictive assumption, static models remain widely used in practice [2]. The first major challenge to this approach came from [3], whose bottleneck model provided a dynamic framework for analyzing peak-period congestion. This approach was later formalized by [4] and further extended by [5]. In the bottleneck framework, travelers trade off congestion delays against schedule delay penalties from arriving earlier or later than their preferred arrival time. Building on this foundation, [6] extended the model to incorporate elastic demand, simple network structures, and user heterogeneity (see [7] and [8], for a comprehensive review).

The behavioral decision of interest in this paper is the choice of departure time, and hence arrival time, under the assumption that travel times are exogenous. This assumption is reasonable in large-scale networks, where the decisions of individual travelers have negligible effects on aggregate traffic conditions. Much of the literature has focused on single-route settings or simplified “toy” networks to study equilibrium outcomes. At equilibrium, since there is a continuum of agents and time is continuous, the generalized cost of using any departure time is equalized across travelers, provided they share identical preferences. However, this is a heroic simplification: in practice, even for a given O–D pair, not all users face the same cost.

In this context, the *α*–*β*–*γ* model [9] has become a standard framework for analyzing scheduling preferences. In this model, *α* denotes the value of travel time, *β* represents the penalty per unit of early arrival, and *γ* the penalty per unit of late arrival. The generalized cost of a trip therefore consists of three components: travel time weighted by *α*, the penalty for early arrival weighted by *β*, and the penalty for late arrival weighted by *γ*. Travelers are assumed to choose their departure (and hence arrival) time to minimize this total cost relative to their preferred arrival time $t^{*}$, which may vary across individuals as do the behavioral parameters *α*, *β*, and *γ*.

Large-scale simulation platforms such as METROPOLIS 1 and 2 [10–12] have incorporated departure-time choice into integrated travel behavior models. These tools simulate users’ learning processes and day-to-day adaptations until a stationary state is reached in which anticipated and experienced travel times converge. The calibration of such models relies critically on behavioral parameters, particularly *α*, *β*, and *γ*. Supply-side parameters, such as road capacity or destination penalties, can be estimated from observed traffic and infrastructure data. In contrast, demand-side scheduling parameters remain difficult to estimate, as they typically require survey data [9, 13].

To the best of our knowledge, [14] represents the only attempt to estimate $\beta /\alpha $ and $\gamma /\alpha $ directly from congestion data. Their approach assumes that any road behaves like a single bottleneck: at equilibrium, the slope of the travel time function identifies $\beta / (\alpha - \beta )$ for early arrivals and $-\gamma /(\alpha +\beta )$ for late arrivals. By contrast, most subsequent studies have relied on stated-preference surveys. For example, Small (1987) estimated a continuous logit model on survey data from 527 U.S. commuters, finding $\beta /\alpha \approx 0.61$ and $\gamma /\alpha \approx 2.38$. This author exploited the fact that departure times constitute an ordered set of alternatives. In a study among seven different cities with survey and traffic data, similar trends have been observed [15]. These estimates have since become standard benchmarks in the literature (see [8]).

While stated-preference surveys have been widely used, they are costly, time-consuming, and limited in scale. Revealed-preference (RP) data—such as traffic counts, travel times, and mode shares—are increasingly abundant thanks to advances in sensing and data collection technologies, including smartphone navigation platforms. This raises a crucial research question: can commuters’ scheduling preferences be reliably inferred from large-scale RP data without relying on surveys? Addressing this question is critical for designing adaptive and data-driven TDM strategies in complex urban environments.

The objective of this paper is to develop a methodology to infer distributions of travelers’ scheduling preferences ($\beta , \gamma $) and desired arrival times $t^{*}$ in dynamic departure-time models from observed arrival and travel-time data. Our approach targets specific population groups, possibly defined by socio-economic attributes and trip purposes, and relies exclusively on attainable RP data, offering a scalable and cost-efficient alternative to survey-based methods. Specifically, the contributions of this study are threefold. First, we propose a structural model that links observed arrival times to scheduling preferences and congestion patterns, characterizing the optimal arrival time as a function of *β*, *γ*, and $t^{*}$. Second, we formulate a likelihood-based estimation framework using maximum likelihood estimation (MLE) to recover the distribution of scheduling parameters directly from RP data. Third, we validate the approach using synthetic data, demonstrating that the method accurately recovers underlying behavioral parameters and can therefore be applied to real-world demand analysis.

The literature has often introduced heterogeneity by assuming that it is observable, typically by segmenting travelers into discrete classes with distinct estimated parameters. In this paper, we adopt a related but more flexible approach, allowing behavioural parameters to vary continuously across individuals. By contrast, [9] treated heterogeneity as unobserved, estimating distributions of scheduling parameters from revealed choices. Our contribution lies in extending the observable-heterogeneity tradition toward continuous estimation, while preserving tractability for estimation purposes.

The rest of the paper is structured as follows. Section 2 characterizes the optimal arrival-time choice problem, given an arbitrary travel-time profile and scheduling preferences. Section 3 formulates the maximum likelihood estimation framework. Section 4 presents numerical experiments based on synthetic data and on field data, while Sect. 5 concludes.

## Analysis on the travel cost of individuals

In this section, we examine the optimal departure problem for individual users under exogenous congestion. First, we introduce the travel cost function assumed for individuals in Sect. 2.1, when the congestion is exogenous and represented by a travel time profile function. The travel cost function turns out to admit multiple local optima, and we analyze the global minimum given the scheduling preference and travel time profile in Sect. 2.2. Based on the analysis, Sect. 2.3 characterizes the optimum explicitly for the travel cost function assuming a representative type of travel time profiles.

### Arrival times and travel costs

Consider a road user commuting between an origin and a destination. The travel time between the origin-destination (OD) pair fluctuates across the time of the day. Let $\mathcal{T} \in \mathbb{R}$ be the set of all possible arrival times. We describe the experienced travel time with a function $tt: \mathcal{T} \rightarrow \mathbb{R}^{+} $. Namely, $tt(t)$ denotes the experienced travel time for travelers arriving at time *t*. To hedge against time-varying congestion, the user may be motivated to arrive early or later at the destinations to avoid the rush hours. To capture such trade-offs, we follow the bottleneck literature by considering that each user chooses an arrival time *t* at their destination that minimizes their individual travel cost.^1^ The travel cost by arriving at any time $t \in \mathcal{T}$ is defined as

1 $$ C(t) = \alpha tt(t) + \beta [t^{*}-t]^{+} + \gamma [t-t^{*}]^{+}, $$

where $t^{*}$ is the desired arrival time at the destination, *α* is the value of travel time, and *β* and *γ* are respectively the unit penalty of arriving early and late at the destination. Without loss of generality, from now on, we normalize the value of *α* to 1. The notations used in this paper are summarized in Table 1 for the convenience of readers.

**Table 1:** Notational Glossary

	Sets
$\mathcal{T}$	Set of all possible arrival times (indexed by *t*)
$\mathcal {E}(t^{*})$/$\mathcal {L}(t^{*})$	Sets of CEA/CLA intervals that contain some desired arrival time $t^{*}$
*E*/*L*	Set of all CEA/CLA intervals
$\mathcal{D}_{conc}$	Interval in which the function *tt* is concave
*Ẽ*/*L̃*	Desired time interval over which it is optimal to arrive early/late
Θ	Set of all possible parameter vectors that characterize a distribution
	Parameters
*tt*	Experienced travel time profile function
$\boldsymbol {t} = (t_{n})_{n = 1, \ldots , N}$	Observed arrival time samples with size *N*
	Indirect Decision Variables
$t^{*}$	Desired arrival time
*α*	Value of travel time
*β*/*γ*	Unit cost of early/late arrival
***δ***	Vector $(\beta , \gamma , t^{*})$
*C*(*t*)	Individual travel cost when arriving at time *t*
$C^{\mathrm{opt}}$	Minimal travel cost
$t^{\mathrm{opt}}$	Arrival time minimzing the individual travel cost
$C^{\mathrm{opt}}_{e}/C^{\mathrm{opt}}_{l}$	Minimal early/late travel cost
$t_{i}^{e}$/$t_{i}^{l}$	Left end-point of an EA/LA interval
$t_{f}^{e}$/$t_{f}^{l}$	Right end-point of an EA/LA interval
$\check{t}_{f}^{e}$/$\check{t}_{i}^{l}$	Right/left end-point of interval *Ě*/ *Ľ*
$\bar{t}^{*}$	Desired arrival time threshold at which $C^{\mathrm{opt}}_{e} = C^{\mathrm{opt}}_{l}$
$\beta _{0}$/$\gamma _{0}$	Threshold of *β*/*γ* beyond which no CEA/CLA interval contains $t^{*}$
$k_{0}$/$k_{1}$	Left/right end-point of $\mathcal{D}_{conc}$
$\beta _{max}$/$\gamma _{max}$	Absolute value of the maximum/minimum derivative of the function *tt*
$\mathcal{L}(\boldsymbol {\theta}; t_{n})$	Likelihood of observing an arrival at time $t_{n}$ when the distribution is parametrized by ***θ***
$f_{t^{\mathrm{opt}}}$/$F_{t^{\mathrm{opt}}}$	PDF/CDF of the observed arrival times
$F_{t^{\mathrm{opt}}}^{o}(t)$	Probability of observing on-time arrivals before time *t*
$F_{t^{\mathrm{opt}}}^{e}$(t)	Probability of observing early arrivals beofre time *t*
$F_{t^{\mathrm{opt}}}^{l}(t)$	Probability of observing late arrivals before time *t*
$f_{\boldsymbol {\delta}}$	PDF of the vector ***δ*** = (*β*,*γ*, $t^{*}$)
	Direct Decision Variables
***θ***	Vector of parameters that characterizes the distribution of ***δ***
$\mu _{\beta}$/$\mu _{\gamma}$/$\mu _{t}$	Mean of *β*/*γ*/$t^{*}$
$\sigma _{t}$	Standard deviation of $t^{*}$
*σ*	Standard deviation of *β* and *γ*

An implied assumption here is that the experienced travel time solely depends on the arrival time, which enables the definition of $tt(t)$. The main reason for this assumption is that $tt(t)$ can be empirically observed with various sensor data, while departure times or $t^{*}$ are not easily observed. The travel time profile is assumed to be exogenous, which is not affected by an individual user’s choices. In addition, we impose a mild assumption regarding the travel time profile function to facilitate our analysis.

#### Assumption 1

The travel time profile function $tt: \mathcal{T} \rightarrow \mathbb{R}^{+} $ is continuous and bounded.

### Minimum of the travel cost function

We characterize the arrival time choices for each individual user with given scheduling preferences parameterized by *β*, *γ*, and the desired arrival time $t^{*}$. Recall that each user chooses the arrival time to minimize their individual travel cost. We define the minimal travel cost $C^{\mathrm{opt}}$ as $C^{\mathrm{opt}} = \min _{t \in \mathcal{T}}C(t)$.

To facilitate our discussion, we introduce two technical terms regarding the conditional minima of the travel cost function $C(t)$: the minimal early arrival cost $C_{e}^{\mathrm{opt}} = \min _{t \le t^{*}}C(t)$ and the minimal late arrival cost $C_{l}^{\mathrm{opt}} = \min _{t \ge t^{*}}C(t)$. Also, we define some special time intervals that relate to our analysis below.

#### Definition 1

Given any travel time profile function *tt* and unit penalty of early and late arrivals *β* and *γ*, an interval $[t_{i}^{e}, t_{f}^{e}]$ with $t_{f}^{e} \ge t_{i}^{e}$ is termed an early arrival (EA) interval when the following condition holds:

2 $$ tt(t ) \ge tt(t_{i}^{e}) + \beta (t - t_{i}^{e} ), \forall t \in [t_{i}^{e}, t_{f}^{e}]. $$

An interval $[t_{i}^{l}, t_{f}^{l}]$ with $t_{f}^{l} \ge t_{i}^{l}$ is termed a late arrival (LA) interval when the following condition holds:

3 $$ tt(t_{f}^{l}) + \gamma (t_{f}^{l} - t ) \le tt(t ), \forall t \in [t_{i}^{l}, t_{f}^{l}]. $$

Intuitively, congestion increases (respectively, decreases) rapidly with the arrival time within each EA (LA) interval, such that the time savings from arriving earlier (later) outweigh the cost of early arrival. We are particularly interested in the intervals that are not contained by others.

#### Definition 2

An EA (respectively, LA) interval is *critical* if it is not strictly contained within any other EA (respectively, LA) interval.

**Figure 1:**
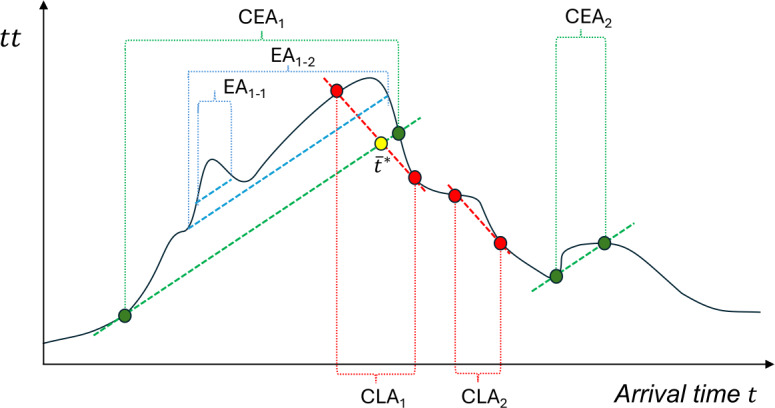
An example of CEA and CLA intervals for a given travel time profile

Figure 1 exemplifies the critical EA(CEA) and LA(CLA) intervals for a general travel time profile *tt*. As we can observe, there are two CEA intervals, CEA_1_ and CEA_2_, and two CLA intervals, CLA_1_ and CLA_2_, in this figure. The first CEA interval CEA_1_ overlaps with the first CLA interval CLA_1_, while the second CEA and CLA intervals, CEA_2_ and CLA_2_, are disjoint. The dashed lines are identified as the indifference curves, whereby users exhibit indifference to arriving at any time within the CEA or CLA intervals, provided that the indifference curves coincide with the travel time profile. Based on Definition 2, a critical early arrival (CEA) interval is simply the largest EA interval that contains a given time point. Note that even though each EA or LA is a continuous interval without interruptions, some EAs (LAs) can contain the others. For example, the EA interval EA_1−1_ is a subinterval of another EA interval EA_1−2_, while EA_1−2_ is a subinterval of the CEA interval CEA_1_.

The introduction of the CEA and CLA intervals allows us to explicitly characterize each individual traveler’s arrival time choice, by characterizing the minima of the travel cost function given the user’s desired arrival time $t^{*}$ and scheduling-preference parameters *β* and *γ*. In particular, given the travel time profile *tt* and a user’s *β* and *γ*, we can identify several special time intervals. By checking whether the user’s desired arrival time $t^{*}$ falls within these intervals, we are able to analyse the user’s minimal travel cost $C^{\mathrm{opt}}$.

#### Proposition 1

*Let* $\mathcal {E}(t^{*})$ *and* $\mathcal {L}(t^{*})$ *denote the sets of CEA and CLA intervals that contain* $t^{*}$, *respectively*. *Under Assumption* 1, *given any* *tt*, *β*, *γ*, *and* $t^{*}$, *when at least one of* $\mathcal {E}(t^{*})$ *and* $\mathcal {L}(t^{*})$ *is empty*, *the optimal travel cost* $C^{\textit{opt}}$, *is given by*

4 $$ C^{\textit{opt}} = \textstyle\begin{cases} tt(t^{*}) , \textit{if } \mathcal {E}(t^{*}), \mathcal {L}(t^{*})= \emptyset , \\ C_{e}^{\textit{opt}}, \textit{if } \mathcal {E}(t^{*}) \neq \emptyset , \mathcal {L}(t^{*})= \emptyset , \\ C_{l}^{\textit{opt}}, \textit{if } \mathcal {E}(t^{*}) = \emptyset , \mathcal {L}(t^{*}) \neq \emptyset . \end{cases} $$

#### Proof

See Appendix A. □

Proposition 1 shows that when the desired arrival time $t^{*}$ does not fall into any CEA or CLA intervals, it is optimal to arrive on time. When $t^{*}$ falls into a CEA interval but not any CLA interval, then the corresponding minimal travel cost is $C^{\mathrm{opt}} = C_{e}^{\mathrm{opt}}$, and it is optimal to arrive early. When $t^{*}$ falls into a CLA interval but not any CEA interval, then the corresponding minimal travel cost is $C^{\mathrm{opt}} = C_{l}^{\mathrm{opt}}$, i.e., it is optimal to arrive late. This follows directly from Lemmas 2 and 3 (Appendix A.1): if both minima reduce to $tt(t^{*})$, being on time wins. If only one improves on $tt(t^{*})$, that dominates. Intuitively, without a “beneficial window”, one has no reason to shift. But if congestion makes one side cheaper, one should shift that way.

Finally, the case when $t^{*}$ falls into both a CEA interval and a CLA interval is discussed in the following proposition.

#### Proposition 2

*Suppose that there exists a CEA interval* $[t_{i}^{e}, t_{f}^{e}]$ *and a CLA interval* $[t_{i}^{l}, t_{f}^{l}]$ *such that* $t^{*} \in [t_{i}^{e}, t_{f}^{e}]$ *and* $t^{*} \in [t_{i}^{l}, t_{f}^{l}]$. *Under Assumption* 1, *there exists a desired arrival time threshold*

5 $$ \bar{t}^{*} = \frac{tt(t_{f}^{l}) - tt(t_{i}^{e}) + \beta t_{i}^{e} + \gamma t_{f}^{l}}{\beta + \gamma},$$

*such that*

6 $$ \textstyle\begin{cases} C_{e}^{\textit{opt}} < C_{l}^{\textit{opt}} , \textit{if } t^{*} < \bar{t}^{*}, \\ C_{e}^{\textit{opt}} = C_{l}^{\textit{opt}}, \textit{if } t^{*} = \bar{t}^{*}, \\ C_{e}^{\textit{opt}} > C_{l}^{\textit{opt}}, \textit{if } t^{*} > \bar{t}^{*}. \end{cases} $$

#### Proof

See Appendix B. □

Proposition 2 demonstrates that, when the desired arrival time $t^{*}$ falls into both a CEA interval and a CLA interval, there exists a threshold of desired arrival time $\bar{t}^{*}$ such that it is optimal to arrive early and late before and after the interval, respectively. Graphically, the indifference curves of early and late arrivals, respectively, intersect with each other at $\bar{t}^{*}$, as shown in Fig. 1. Early and late costs are exactly equal at $\bar{t}^{*}$. As $t^{*}$ increases, early arrival costs rise (penalty *β* applies), late arrival costs fall (penalty *γ* applies). Therefore, one side dominates before and the other after. Thus, if someone’s desired arrival time is earlier than the “crossover”, she will lean toward arriving early; if later, arriving late is cheaper. The threshold is like a pivot point. Note that the threshold $\bar{t}^{*}$ is not necessarily in the CEA or CLA interval when one of the intervals contains the other.

So far, we are able to identify the global minima of any user’s travel cost function given her $t^{*}$, and CEA and CLA intervals. However, usually we do not know each user’s CEA and CLA intervals, but her *β* or *γ*. Thus, we continue to analyze how each user’s *β*/*γ* affect her CEA/CLA intervals, as formalized in the following proposition.

#### Proposition 3

*Let* $[t_{i}^{e}, t_{f}^{e}]$ *and* $[t_{i}^{l}, t_{f}^{l}]$ *be a CEA interval and a CLA interval*, *respectively*, *under some* *β* *and* *γ*. *Under Assumption* 1, $t_{i}^{e}$ *and* $t_{f}^{e}$ *are weakly increasing and decreasing in* *β*, *respectively*, *and* $t_{i}^{l}$ *and* $t_{f}^{l}$ *are weakly increasing and decreasing in* *γ*, *respectively*.

#### Proof

See Appendix C. □

We show in Proposition 3 that, when *β* (respectively, *γ*) increases, each CEA (respectively, CLA) interval will shrink. That is, a larger *β* means early arrivals are more painful, so the range of profitable early arrivals shrinks. Similarly, for *γ* and late arrivals. Intuitively, if someone hates being early (high *β*), the window where “arriving early to dodge congestion” makes sense becomes narrower. The intuition applies to late arrivals as well. Proposition 3 directly leads to the following corollary.

#### Corollary 1

*Under Assumption* 1, *given any desired arrival time* $t^{*}$, *there exists an threshold* $\beta _{0}$ *of early*-*arrival penalty such that* $t^{*}$ *is not in any CEA interval*, *i*.*e*., $\mathcal {E}(t^{*}) = \emptyset $, *if and only if* $\beta > \beta _{0}$. *Also*, *there exists a threshold of late*-*arrival penalty* $\gamma _{0}$ *such that* $t^{*}$ *is not in any CLA interval*, *i*.*e*., $\mathcal {L}(t^{*}) = \emptyset $, *if and only if* $\gamma > \gamma _{0}$.

Corollary 1 shows that for each $t^{*}$, there is a threshold $\beta _{0}$ (or $\gamma _{0}$) beyond which no CEA (or CLA) interval contains $t^{*}$. This follows from Proposition 3: as *β* increases to infinity, eventually no early window is worthwhile. The same logic applies to *γ*. By Corollary 1, we can conveniently check if a user’s $t^{*}$ falls into any of her CEA or CLA intervals by knowing her *β* or *γ*, respectively.

**Figure 2:**
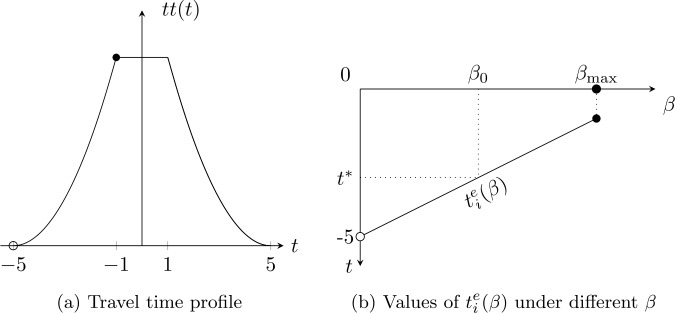
An example with an idealized travel time profile function on the impact of *β* on the left endpoint $t_{i}^{e}(\beta )$ of a CEA interval

Figure 2 exemplifies the threshold in an idealized case. The travel time profile function is $tt(t) = (5 - |t| )^{2}$ for any *t* in $[-5, -1] $ and $[1,5]$. The left endpoint $t_{i}^{e}(\beta )$ of the CEL interval is linearly increasing in *β* when $\beta < \beta _{max}$. When $\beta =0$, the time interval $[-5, 5]$ is a CEA interval, and thus any $t^{*} \in [-5, 5]$ falls into the CEA interval. When $\beta > \beta _{max}$, there is no CEA interval. When $\beta \in (0, \beta _{max})$, the CEA interval exists and its left endpoint $t_{i}^{e}$ is always smaller than −1, and the right endpoint is always greater than −1. Thus, if $t^{*} \in [-5, -1]$, the threshold $\beta _{0}$ is such that $t_{i}^{e}(\beta _{0}) = t^{*} $; if $t^{*} \in [-1, 5]$, the threshold $\beta _{0}$ is such that $t_{f}^{e}(\beta _{0}) = t^{*} $. The threshold $\gamma _{0}$ can be analogously analyzed for late arrivals.

### Convex-concave-convex travel time profiles

Now, we continue our analysis by considering a representative congestion pattern in which the travel time profile exhibits a convex–concave–convex shape, as described by the following assumption.

#### Assumption 2

The travel time profile function *tt* is unimodal and twice differentiable. Furthermore, there exists a time interval $\mathcal{D}_{conc} = [k_{0}, k_{1}]$ such that $tt'' \le 0$ in $\mathcal{D}_{conc}$, and $tt'' \ge 0$ in $\mathcal{D}_{conv} = \mathcal{T} \setminus \mathcal{D}_{conc}$.

**Figure 3:**
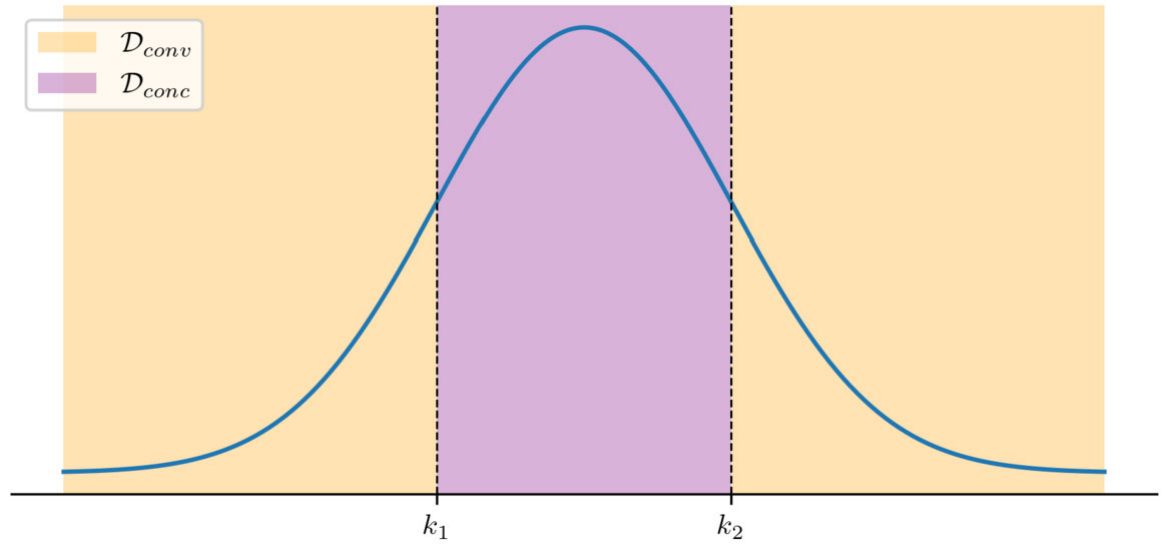
Example of a travel time profile under Assumption 2. The shaded zones show the parts of its domain in which the function is convex or concave

Under Assumption 2, congestion will monotonically increase during the day up until a certain time, and then monotonically decrease afterwards, as shown in Fig. 3. Additionally, it requires the derivative of the travel time to be unimodal both before and after the peak. This assumption is moderate because we allow for different sizes of $\mathcal{D}_{conc}$ and $\mathcal{D}_{conv}$ such that the curve can approximate most real travel time profiles in practice.

In this case, we can explicitly characterize the endpoints of the CEA and CLA intervals, as formalized below.

#### Proposition 4

*Under Assumptions* 1 - 2, *there exist at most one CEA interval* $[t_{i}^{e}, t_{f}^{e}]$ *and at most one CLA interval* $[t_{i}^{l}, t_{f}^{l}]$ *such that* $tt'(t_{i}^{e})= \beta $ *and* $tt'(t_{f}^{l})= \gamma $.

#### Proof

See Appendix D. □

Proposition 4 characterizes the left and right endpoints of the CEA and CLA intervals (if they exist), respectively, under unimodal, smooth travel time profiles described by Assumption 2. The unimodal shape ensures derivative crosses *β* (or *γ*) at most once. Thus, only one critical interval of each type exists. With a single congestion peak, there’s at most one useful “early zone” and one “late zone”. The slope conditions capture exactly when congestion growth/decline offsets penalties.

**Figure 4:**
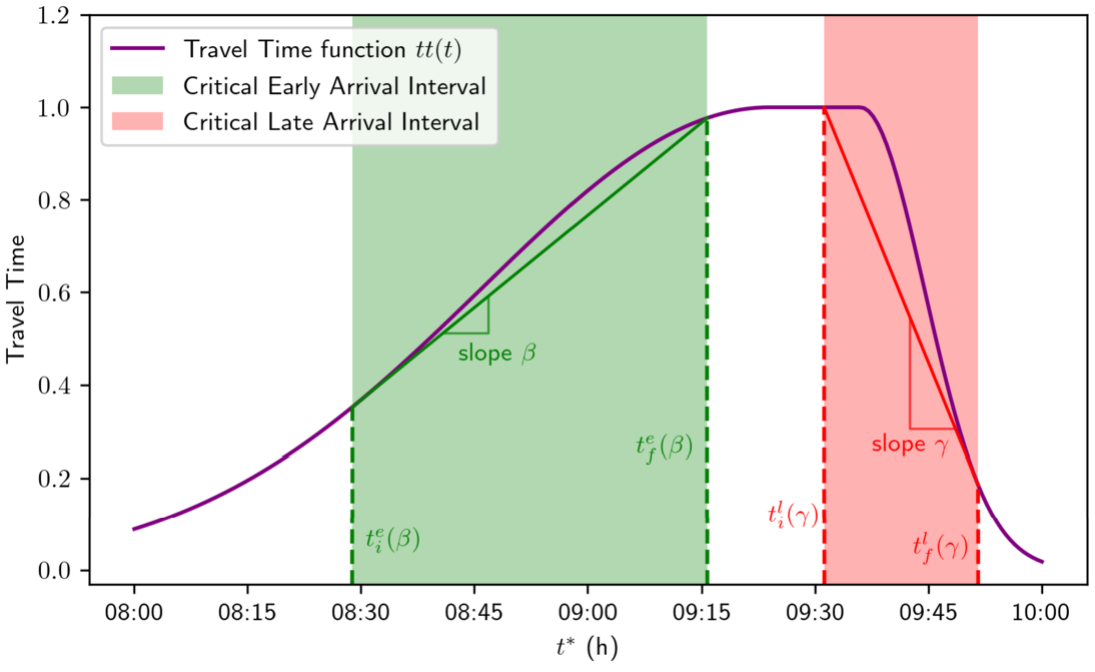
Critical early and late arrival intervals for a travel time profile under Assumption 2 given *β* and *γ*

By definitions of CEA intervals, at the right endpoint $t_{f}^{e}$ of the CEA interval, the line tangent to the travel time function at $t_{f}^{e}$ (that is, the green solid line in Fig. 4) intersects with the travel time profile. Symmetrically, at $t_{i}^{l}$, the red solid line intersects with the travel time profile. Also, the red and the green solid lines intersect at $\bar{t}^{*}$, as defined in Proposition 2.

By Proposition 2, if both the CEA interval $[t_{i}^{e}, t_{f}^{e}]$ and the CLA interval $[t_{i}^{l}, t_{f}^{l}]$ exist under Assumption 2, we can futher identify two intervals *Ẽ*, and *L̃* of the desired arrive time $t^{*}$: $\tilde{E} = [t_{i}^{e}, \check{t}_{f}^{e} ]$, $\tilde{L} = [\check{t}_{i}^{l}, t_{f}^{l}] $, where $\check{t}_{f}^{e} = \min \{t_{f}^{e}, \bar{t}^{*}\}$, and $\check{t}_{i}^{l}(\gamma ) = \max \{t_{i}^{l}, \bar{t}^{*}\} $. That is, it is optimal to arrive early and late if a user’s $t^{*}$ falls into *Ẽ* and *L̃*, respectively.

#### Corollary 2

*Under Assumptions* 1 - 2, *given any* *tt*, *β*, *γ*, *and* $t^{*}$, *the optimal travel cost* $C^{\textit{opt}}$, *is given by*

7 $$ C^{\textit{opt}} = \textstyle\begin{cases} C_{e}^{\textit{opt}}, \textit{if } t^{*} \in \tilde{E}, \\ C_{l}^{\textit{opt}}, \textit{if } t^{*} \in \tilde{L}, \\ tt(t^{*}), \textit{otherwise,} \end{cases} $$

*where* $\tilde{E} = [t_{i}^{e}, \check{t}_{f}^{e} ]$, $\tilde{L} = [\check{t}_{i}^{l}, t_{f}^{l}]$ *with* $\check{t}_{f}^{e} = \min \{t_{f}^{e}, \bar{t}^{*}\}$, *and* $\check{t}_{i}^{l} = \max \{t_{i}^{l}, \bar{t}^{*}\} $.

Corollary 2 is a direct combination of Propositions 1-4. By Corollary 2, under Assumptions 1–2, the optimal arrival cost is early, late, or on-time depending on whether $t^{*}$ falls inside the unique reduced CEA/CLA zones. Intuitively, the framework gives a clean rule: each user’s optimal arrival strategy depends entirely on whether her desired time sits in the ‘profitable’ early or late windows.

Let $\beta _{max} = \max _{t} tt'(t)$ and $\gamma _{max} = -\min _{t} tt'(t)$, respectively. When a traveler has a large early arrival penalty, i.e., $\beta > \beta _{max}$, the set *Ẽ* is empty, and therefore it is never optimal to arrive early regardless of *γ* and $t^{*}$. Symmetrically, when $\gamma > \gamma _{max}$, it is never optimal to arrive late regardless of *β* and $t^{*}$.

So far, we have established the optimal arrival time choices of an individual given exogenous travel time profile function *tt*, and scheduling preferences characterized by *β*, *γ*, and $t^{*}$. When we consider a population with heterogeneous *β*, *γ*, and $t^{*}$, the resulting arrival time choices are distributed. When the distribution of *β*, *γ*, and $t^{*}$ among the population is given, we can further derive the distribution of observed arrival times among the population. Obviously, heterogeneity does not add much to the complexity of our analysis. This analysis will essentially yield a structural model describing the heterogeneous arrival time choices in peak hours in the following section, which can be calibrated by the maximum likelihood estimate method.

## Maximum likelihood estimation

In this section, we begin by introducing the Maximum Likelihood Estimation (MLE) problem in Sect. 3.1. Then, in Sect. 3.2, we explicitly characterize the distribution of the optimal arrival times, characterized by its probability density function (PDF), when the distribution of *β*, *γ*, and $t^{*}$ among the population is given.

### Problem statement

**Figure 5:**
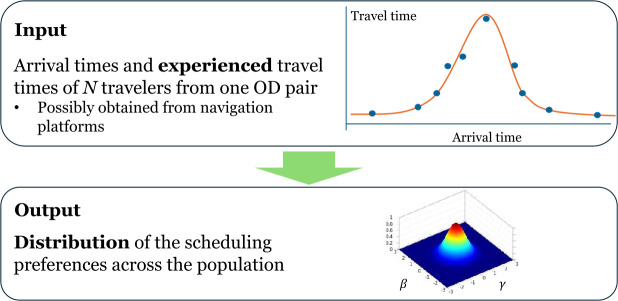
The main objective of this paper

We consider an authority aiming to ascertain the preferences of a population of travelers who exhibit heterogeneous scheduling preferences for commuting between an origin and destination (OD) pair. A transportation authority would like to know the distribution of their scheduling preferences, which are characterized by a vector $\boldsymbol {\delta}= (\beta , \gamma , t^{*})$, but it is quite challenging to conduct a survey on that. However, the authority has the data regarding (1) the experienced travel time profile *tt* for travelers arriving at different times of the day, and (2) the observations of arrival times for a certain number of travelers. Our goal is to estimate the distribution of ***δ***, as shown in Fig. 5.

Formally, let Θ denote the set of all possible parameter vectors that characterize a distribution of ***δ*** across the population, and $\boldsymbol {t} = (t_{n})_{n = 1, \ldots , N}$ denote the observed arrival time samples, where *N* is the number of observations. We aim to find a vector $\boldsymbol {\theta}^{*} \in \Theta $ that maximizes the likelihood of the observed data ***t***, i.e.,

8 $$ \boldsymbol {\theta}^{*} = \operatorname*{argmin}_{\boldsymbol {\theta}\in \Theta} \prod _{\boldsymbol {t}} \mathcal{L}(\boldsymbol {\theta}; t_{n}), $$

where $\mathcal{L}(\boldsymbol {\theta}; t_{n})$ is the likelihood of observing arrival time $t_{n}$ when the distribution of ***δ*** is parametrized by ***θ***.

### Likelihood

Given the joint distribution of *β*, *γ*, and $t^{*}$ characterized by ***θ***, the likelihood of observing an arrival at time *t* is the probability density of $t^{\mathrm{opt}}$:

9 $$ \mathcal{L}(\boldsymbol {\theta}; t) = f_{t^{\mathrm{opt}}}(t; \boldsymbol {\theta}), $$

where $f_{t^{\mathrm{opt}}}$ is the probability density of observed arrival times. When the joint probability density $f_{\boldsymbol {\delta}}$ of $\boldsymbol {\delta}= (t^{*}, \beta , \gamma )$ is known, we are able to derive the expression of $f_{t^{\mathrm{opt}}}$ as below.

Each observed arrival can be either on-time, early, or late, of which the probabilities are discussed separately as follows. Let $F_{t^{\mathrm{opt}}}(t)$ denote the CDF of the observed arrival times. We have

10 $$ F_{t^{\mathrm{opt}}}(t) = F_{t^{\mathrm{opt}}}^{o}(t) + F_{t^{\mathrm{opt}}}^{e}(t) + F_{t^{\mathrm{opt}}}^{l}(t), $$

where $F_{t^{\mathrm{opt}}}^{o}(t)$, $F_{t^{\mathrm{opt}}}^{o}(t)$, and $F_{t^{\mathrm{opt}}}^{o}(t)$ are probabilities of observing arrivals at any time $t^{\mathrm{opt}} < t$ and the arrives being on-time, early, or late, respectively. Namely, $F_{t^{\mathrm{opt}}}^{o}(t) = \mathbb{P}(t^{\mathrm{opt}} < t; C^{ \mathrm{opt}}_{l} =C_{e}^{\mathrm{opt}})$, $F_{t^{\mathrm{opt}}}^{e}(t) = \mathbb{P}(t^{\mathrm{opt}} < t; C^{ \mathrm{opt}}_{l} > C_{e}^{\mathrm{opt}})$, and $F_{t^{\mathrm{opt}}}^{l}(t) = \mathbb{P}(t^{\mathrm{opt}} < t; C^{ \mathrm{opt}}_{l} < C_{e}^{\mathrm{opt}})$. For simplicity, we consider in the following discussion that the travel time profile function *tt* satisfies Assumption 2.

#### On-time arrivals

By Corollary 2, an arrival at time *t* is on-time (i.e., $C^{\mathrm{opt}}_{l} = C_{e}^{\mathrm{opt}}$) if and only if the observed user’s desired arrival time $t^{*}$ does not fall into intervals *Ẽ* and *L̃*. Thus, the probability of observing arrivals at any time $t^{\mathrm{opt}} < t$ and the arrivals being on-time is given by

11 $$ \begin{aligned} F_{t^{\mathrm{opt}}}^{o}(t) & = \mathbb{P}(t^{\mathrm{opt}} \le t, t^{*} \notin \tilde{E}(\beta , \gamma ) \cup \tilde{L}(\beta , \gamma )). \end{aligned} $$

Using Corollary 1, we can conclude that under Assumptions 1 - 2, given any $t^{*}$, there exists a threshold $\beta _{0}(t^{*})$ of *β* such that $t^{*} \notin \tilde{E}(\beta , \gamma )$ when $\beta > \beta _{0}(t^{*})$, and a threshold $\gamma _{0}(t^{*})$ such that $t^{*} \notin \tilde{L}(\beta , \gamma )$ when $\gamma > \gamma _{0}(t^{*})$. Therefore we have

12 $$ \begin{aligned} F_{t^{\mathrm{opt}}}^{o}(t) & = \mathbb{P}(t^{*} \le t, \beta > \beta _{0}(t^{*}), \gamma > \gamma _{0}(t^{*})) \\ & = \int _{0}^{t} \int _{\beta _{0}(\tau )}^{\infty}\int _{\gamma _{0}( \tau )}^{\infty} f_{\boldsymbol {\delta}}(\tau , b , g)\, dg\, db\, d\tau . \end{aligned} $$

Note that the corresponding probability density is $f_{t^{\mathrm{opt}}}^{o}(t) = \partial F_{t^{\mathrm{opt}}}^{o}(t)/ \partial t$. Therefore, the probability density is

13 $$ \begin{aligned} f_{t^{\mathrm{opt}}}^{o}(t) &= \int _{\beta _{0}(t)}^{ \infty}\int _{\gamma _{0}(t)}^{\infty} f_{\boldsymbol {\delta}}(t, b , g)\, dg \, db. \end{aligned} $$

#### Early arrivals

By Corollary 2, an observed arrival at time *t* is early (i.e., $C^{\mathrm{opt}} = C^{\mathrm{opt}}_{e}$) when the observed user’s preference parameters *β* and *γ* are such that her desired arrival time $t^{*}$ falls into the interval $\tilde{E}(\beta , \gamma )$. Then we have

14 $$ \begin{aligned} F_{t^{\mathrm{opt}}}^{e}(t) &= \mathbb{P}(t^{\mathrm{opt}} \le t, C^{\mathrm{opt}} = C_{e}^{\mathrm{opt}}) \\ & = \mathbb{P}(t^{\mathrm{opt}} \le t, t^{*} \in \tilde{E}(\beta , \gamma ) ). \end{aligned} $$

Equivalently, the event $t^{\mathrm{opt}} \le t$ and $C^{\mathrm{opt}}= C_{e}^{\mathrm{opt}}$ occurs if and only if the left endpoint $t_{i}^{e}(\beta )$ of $\tilde{E}(\beta , \gamma )$ is not greater than time *t* (i.e., $t_{i}^{e}(\beta ) \le t$), and $t^{*}$ falls into interval $\tilde{E}(\beta , \gamma ) $. Recall that by Assumption 2, the travel time profile function *tt* is convex when $t \le k_{0}$ or $t \ge k_{1}$, and concave when $t \in [k_{0}, k_{1}]$. We proceed with our analysis in two cases separately:

Case 1: $t \le k_{0}$, i.e., $tt''(t) \ge 0 $ and $tt'>0$. By Proposition 3, the left endpoint $t_{i}^{e}(\beta )$ of *Ẽ* is weakly increasing in *β* when $\beta \le \beta _{max}$. In this case, there exists a threshold $\bar{\beta}= tt'(t)$ of *β* such that $t_{i}^{e}(\beta )$ is not greater than time *t*, i.e., $t_{i}^{e}(\beta ) \le t$, when *β* is not greater than $tt'(t)$. We have

15 $$ \begin{aligned} F_{t^{\mathrm{opt}}}^{e}(t) &= \mathbb{P}(\beta \le tt'(t), t^{*} \in \tilde{E}(\beta , \gamma ) ) \\ & = \int _{0}^{tt'(t)} \int _{0}^{\infty }\int _{t_{i}^{e}(b)}^{ \check{t}_{f}^{e}(b, g) } f_{\boldsymbol {\delta}}(\tau , b , g) d\tau d g d b. \end{aligned} $$

Case 2: $t > k_{0}$. When $\beta \le \beta _{max}$, the interval *Ẽ* exists, of which the left-end point $t_{i}^{e}(b)$ is always not greater than *t*. Then we have

16 $$ \begin{aligned} F_{t^{\mathrm{opt}}}^{e}(t) &= \mathbb{P}(\beta \le \beta _{max}, t^{*} \in \tilde{E}(\beta , \gamma ) ) \\ & = \int _{0}^{\beta _{max}} \int _{0}^{\infty }\int _{t_{i}^{e}(b)}^{ \check{t}_{f}^{e}(b, g) } f_{\boldsymbol {\delta}}(\tau , b , g) d\tau d g d b. \end{aligned} $$

Again, the probability density $f_{t^{\mathrm{opt}}}^{e}(t) = \partial F_{t^{\mathrm{opt}}}^{e}(t) / \partial t$ can be expressed as

17 $$ f_{t^{\mathrm{opt}}}^{e}(t) = \textstyle\begin{cases} tt''(t) \int _{0}^{\infty }\int _{t_{i}^{e}(tt'(t))}^{\check{t}_{f}^{e}(tt'(t), g) } f_{\boldsymbol {\delta}}(\tau , tt'(t), g) d\tau d g, \text{if } t \le k_{0}, \\ 0, \mathrm{otherwise}. \end{cases} $$

Note that we have $t_{i}^{e}(tt'(t)) = t$ when $t \le k_{0}$ by definition. Moreover, since *β* is nonnegative, we have $f_{\boldsymbol {\delta}}(\tau , \beta , g) = 0$ when $\beta = tt'(t) <0$. Then we can simplify the above expression of $f_{t^{\mathrm{opt}}}^{e}(t)$ as

18 $$ f_{t^{\mathrm{opt}}}^{e}(t) =[tt''(t)]^{+} \int _{0}^{\infty }\int _{t}^{ \check{t}_{f}^{e}(tt'(t), g) } f_{\boldsymbol {\delta}}(\tau , tt'(t), g) d\tau d g. $$

#### Late arrivals

The analysis for the late arrivals is similar to that for the early arrivals. Similarly, we have

19 $$ \begin{aligned} F_{t^{\mathrm{opt}}}^{l}(t) &= \mathbb{P}(t^{\mathrm{opt}} \le t, C^{\mathrm{opt}} = C_{e}^{\mathrm{opt}}) \\ & = \mathbb{P}(t^{\mathrm{opt}} \le t, t^{*} \in \tilde{L}(\beta , \gamma ) ). \end{aligned} $$

Equivalently, the event $t^{\mathrm{opt}} \le t$ and $C^{\mathrm{opt}} = C_{l}^{\mathrm{opt}}$ occurs if and only if the right endpoint $t_{f}^{l}(\beta )$ of $\tilde{L}(\beta , \gamma ) $ is not greater than time *t*, and $t^{*}$ falls into interval $\tilde{L}(\beta , \gamma ) $. Again, we have

20 $$ \begin{aligned} F_{t^{\mathrm{opt}}}^{l}(t) &= \mathbb{P}(\gamma \le -tt'(t), t^{*} \in \tilde{L}(\beta , \gamma ) ) \\ & = \int _{0}^{-tt'(t)} \int _{0}^{\infty }\int _{t_{f}^{l}(g)}^{ \check{t}_{i}^{l}(b, g) } f_{\boldsymbol {\delta}}(\tau , b , g) d\tau d b d g , \end{aligned} $$

if $t \ge k_{1}$; otherwise

21 $$ \begin{aligned} F_{t^{\mathrm{opt}}}^{l}(t) &= \mathbb{P}(\gamma \le -tt'(t), t^{*} \in \tilde{L}(\beta , \gamma ) ) \\ & = \int _{0}^{\gamma _{max}} \int _{0}^{\infty }\int _{t_{f}^{l}(g)}^{ \check{t}_{i}^{l}(b, g) } f_{\boldsymbol {\delta}}(\tau , b , g) d\tau d b d g. \end{aligned} $$

Note that the corresponding probability density is $f_{t^{\mathrm{opt}}}^{l}(t) = \partial F_{t^{\mathrm{opt}}}^{l}(t)/ \partial t$. Again, we have

22 $$ f_{t^{\mathrm{opt}}}^{l}(t) = \textstyle\begin{cases} - tt''(t) \int _{0}^{\infty }\int _{t_{f}^{l}(- tt'(t))}^{\check{t}_{i}^{l}(b, - tt'(t)) } f_{\boldsymbol {\delta}}(\tau , b, - tt'(t)) d\tau d g, \text{if } t > k_{1}, \\ 0, \mathrm{otherwise}. \end{cases} $$

Therefore, we obtain that

23 $$ f_{t^{\mathrm{opt}}}^{l}(t) = [- tt''(t)]^{+} \int _{0}^{\infty }\int _{t}^{ \check{t}_{i}^{l}(b, - tt'(t)) } f_{\boldsymbol {\delta}}(\tau , b, - tt'(t)) d \tau d b. $$

#### Independent *β*, *γ* and $t^{*}$

Without loss of much generality, we can further consider that *β*, *γ*, and $t^{*}$ are independently distributed for simplicity.

##### Assumption 3

The variables *β*, *γ*, and $t^{*}$ are independently distributed among the population.

Let $f_{\beta}(\beta )$, $f_{\gamma}(g)$, and $f_{t^{*}}(t^{*})$ denote the probability density functions of *β*, *γ*, and $t^{*}$, respectively. By Equation (10), we have $f_{t^{\mathrm{opt}}}(t) = f_{t^{\mathrm{opt}}}^{o}(t) + f_{t^{\mathrm{opt}}}^{e}(t) + f_{t^{\mathrm{opt}}}^{l}(t)$. Therefore, under Assumption 3, we obtain

24 $$ \begin{aligned} f_{t^{\mathrm{opt}}}(t) & = f_{t^{*}}(t) \int _{\beta _{0}(t)}^{ \infty} f_{\beta}(b) \int _{\gamma _{0}(t)}^{\infty} f_{\gamma}(g) \, dg \, db \\ & + [tt''(t)]^{+} f_{\beta}(tt'(t)) \int _{0}^{\infty }f_{\gamma}(g) \int _{t}^{\check{t}_{f}^{e}(tt'(t), g) } f_{t^{*}}(t) d\tau d g \\ &+ [- tt''(t)]^{+} f_{\gamma}(- tt'(t)) \int _{0}^{\infty }f_{\beta}(b) \int _{t}^{\check{t}_{i}^{l}(b, - tt'(t)) } f_{t^{*}}(t) d\tau d b. \end{aligned} $$

So far, we have explicitly characterized the distribution of the optimal arrival times $t^{\mathrm{opt}}$ by the above PDF $f_{t^{\mathrm{opt}}}(t;\boldsymbol {\theta})$ under any distribution of $\boldsymbol {\delta}= (\beta , \gamma , t^{*})$ parameterized by ***θ***. Apparently, the MLE problem defined in (9) is a nonlinear and nonconvex optimization problem, which is difficult to solve exactly in general. To this end, we develop our own solution methods as detailed below.

## Methods for solving the MLE problem

In this section, we elaborate on the methods developed to solve the MLE problem defined in (8) efficiently. Aside from being nonlinear and nonconvex in general, the likelihood in the objective function of Problem (8) is expressed in a convoluted way such that it is difficult to derive the gradient explicitly. We resort to gradient-free optimization techniques for computational stability, as detailed in Sect. 4.1. We further explain how the likelihood is evaluated within the proposed solution framework in Sect. 4.2.

### Optimization framework

Since the likelihood function in our MLE problem involves numerous integrals, with some upper limits lacking closed-form expressions, direct gradient-based approaches such as implicit differentiation methods (e.g., [16]) are not applicable. To address this challenge, we employ a two-step optimization strategy, described as follows.

**Figure 6:**
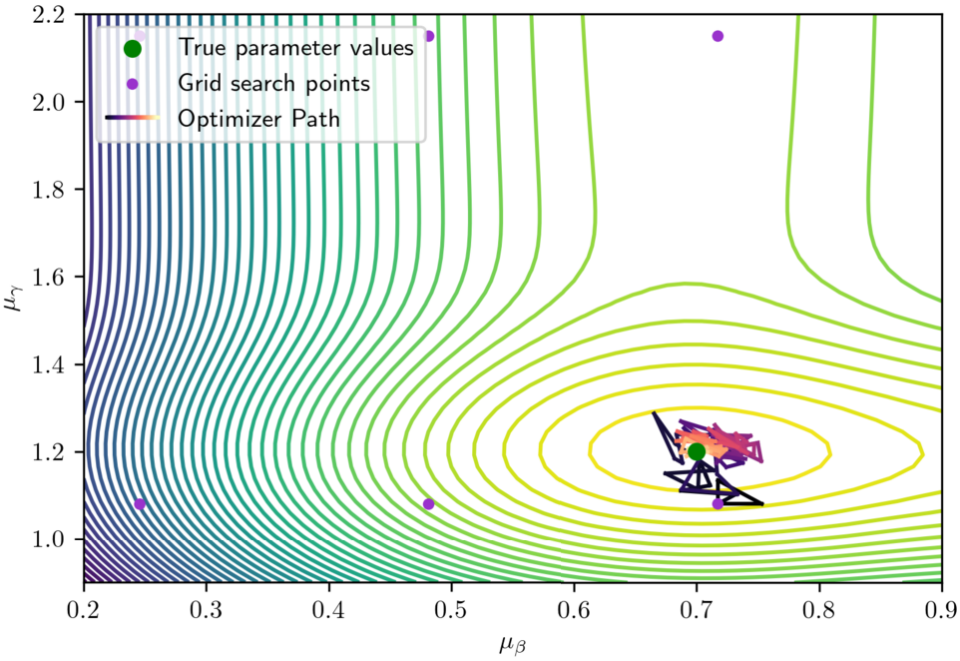
An example of the realization of the optimization framework

We adopt a two-step framework to solve the problem. First, we conduct a grid search to find an initial solution for the optimizer. Second, we employ the Nelder-Mead optimizer [17] which avoids computing gradients. The Nelder–Mead optimizer is a derivative-free numerical algorithm that minimizes a function by iteratively updating a simplex of points to explore and converge toward the optimum. As shown in Fig. 6, the contour plot shows the objective function $\prod _{\boldsymbol {t}} \mathcal{L}(\boldsymbol {\theta}; t_{n})$ of Problem (8), i.e., the likelihood, under different $\mu _{\beta}$ and $\mu _{\gamma}$. The initial point is first found through the grid search, depicted in purple. Then the Nelder-Mead optimizer further maximizes the objective function and converges to the optimum. We are typically able to reach convergence, with relative errors around 5%, in about 150 iterations. That is, the proposed optimization framework achieves a sufficiently precise convergence in this example.^2^

### Computation of the likelihood

The objective function of Problem (8), comprised of the likelihood in (24), is computationally expensive. When applying the Nelder–Mead method to maximize the likelihood, each iteration requires evaluating the likelihood function in (24) at multiple parameter values. Since the likelihood in our setting involves several integrals—some with non-closed-form upper and lower limits—this procedure demands efficient and accurate computation of these integrals since the likelihood of thousands of points has to be estimated; otherwise, the optimization would become prohibitively slow or numerically unstable. We therefore develop specialized techniques for the evaluation. Interested readers are referred to Appendix E for more details.

## Numerical experiment with synthetic data

In this section, we conduct numerical experiments to evaluate the proposed method using synthetic data. The procedure is organized as follows. First, we prepare the input for the experiment in Sect. 5.1, including the specification of a theoretical travel time function in Sect. 5.1.1 and the distributions of the parameters *β*, *γ*, and $t^{*}$ in Sect. 5.1.2. Next, we generate a synthetic dataset by sampling *β*, *γ*, $t^{*}$, explicitly minimizing the resulting travel cost function, and obtaining observations of arrivals in Sect. 5.2. We then compare the sampled data with the theoretical predictions in Sect. 5.2.2. Finally, we evaluate the performance of the MLE method in recovering the true parameters of the distributions in Sect. 5.3.

### Input data

#### Travel time profile function

The travel time profile function adopted in this section is a synthesis of two Gaussian distributions. To better represent the asymmetry for early and late arrivals, two different variances will be considered before and after the peak. The considered function is

25 $$ tt_{g}(t) = \textstyle\begin{cases} e^{-\frac{(x - \mu )^{2}}{\sigma _{l}}}, & \text{if } x \leq \mu , \\ e^{-\frac{(x - \mu )^{2}}{\sigma _{r}}}, & \text{if } x > \mu . \end{cases} $$

**Figure 7:**
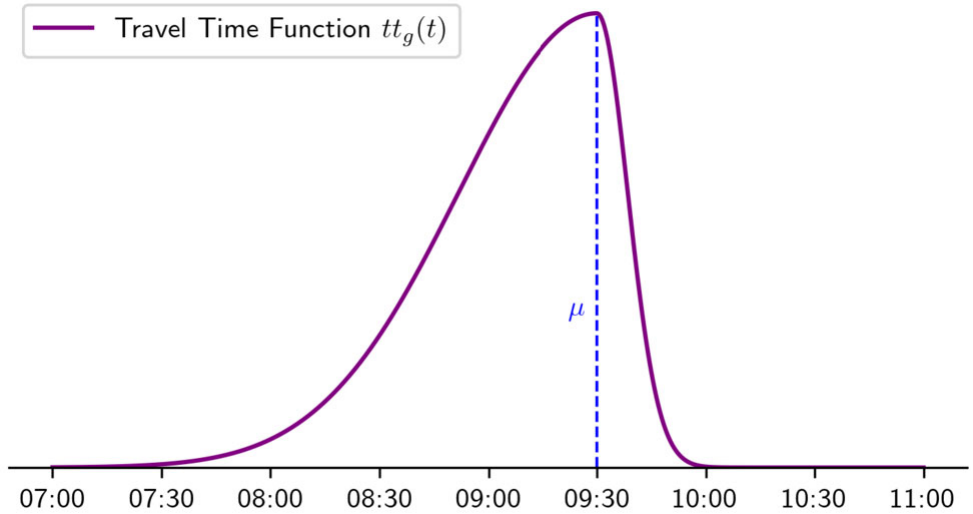
Theoretical travel time function $tt_{g}(t)$ as defined in (25)

The chosen travel time function is bounded and continuous, with a differentiable derivative, and it satisfies the convexity and concavity conditions specified in Assumption 2. Hence, the function fulfills both Assumptions 1 and 2. We plot the function in Fig. 7. The parameter $\sigma _{l}$, regulating the steepness before the peak, is higher than the parameter $\sigma _{r}$, which regulates the steepness after the peak.

#### Distribution of the preference parameters

For simplicity, we assume that *β*, *γ*, and $t^{*}$ are normally distributed, with *β* and *γ* sharing the same variance:

$$ \beta \sim \mathcal{N}(\mu _{\beta}, \sigma ^{2}), \quad \gamma \sim \mathcal{N}(\mu _{\gamma}, \sigma ^{2}), \quad t^{*} \sim \mathcal{N}( \mu _{t}, \sigma _{t}^{2}). $$

The chosen distribution trivially satisfies assumption 3, and therefore we can directly employ Equation (24). This specification of the distributions naturally determines the parameter vector

$$ \boldsymbol {\theta}= (\mu _{\beta}, \mu _{\gamma}, \mu _{t}, \sigma , \sigma _{t})^{T} \in \mathbb{R}^{5}. $$

In the following, the values of *θ* will be chosen in order to adhere as much as possible to the existing literature [18], while preserving the identifiability of the developed model.

### Generating arrivals

To generate input data on the observed arrival times, we sample a population of size *N*, where each individual’s parameters *β*, *γ*, and $t^{*}$ are drawn from a prior distribution selected in Sect. 5.1.2. This yields a dataset of triples $\{\beta _{i}, \gamma _{i}, t^{*}_{i}\}_{i}$, where $i = 1, \ldots , N$. For each sampled user *i*, we simulate her actual arrival time $t_{i}$ by minimizing her travel cost function, i.e., $t_{i} = \operatorname*{argmin}_{t} C(t; \beta _{i}, \gamma _{i}, t^{*}_{i}) $.

#### Algorithm of minimizing the travel cost function

Under Assumption 2, the travel cost function admits at most three local optima. Two of them occur before and after the desired arrival time $t^{*}$, while the third is located at $t^{*}$. By Equation (1), the travel cost function is not differentiable at $t^{*}$, but differentiable at the other two local optima.

To address this, we calculate the three local optima separately. The first two are obtained using gradient descent methods, initialized at an early time (e.g., $t=0$) and a late time (e.g., $t=24$). We then compare the values of the objective function at these two local optima with the value achieved at $t = t^{*}$.

**Figure 8:**
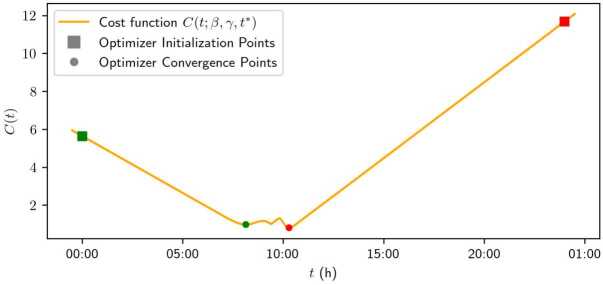
Travel cost function with $\beta = 0.6$ and $\gamma = 0.8$

Figure 8 shows the travel cost function and illustrates the optimization process. The squares represent the optimizer initialization, and the dots indicate the minima to which the optimizers converge. The optimizer initialized in green converges to the early minimum, while the one in red converges to the late minimum. By comparing these minima with the cost of arriving on time, the global minimizer of the cost function is identified.

#### Comparing sampled arrivals with theoretical likelihood

**Figure 9:**
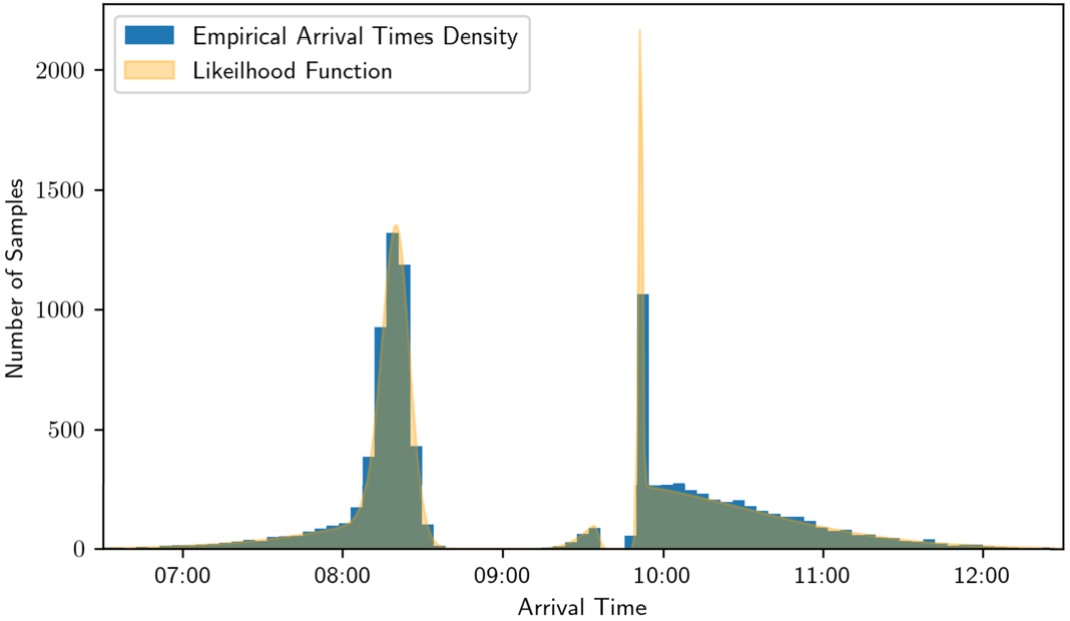
Histogram displaying the distribution of $N=10{,}000$ sampled arrival times, plotted over the result of the likelihood function in Expression (24)

Finally, the generated arrivals for the sampled users are illustrated in Fig. 9. The parameters for the distributions are $\boldsymbol {\theta}_{0} = (\mu _{\beta}, \mu _{\gamma}, \mu _{t}, \sigma, \sigma _{t})^{T} = (0.6, 2.4, 9.5, 0.1, 1)^{T}$, and the travel time function $tt_{g}$ defined in (25) has coefficients $\mu = 9.5$, $\sigma _{l} = 0.9$, and $\sigma _{r} = 0.2$. The dashed lines indicate the points where the derivative of the travel time function equals the mean of the scheduling delay preferences, which, as expected, correspond to the regions of higher density.

We show in Fig. 9 that the theoretical density closely matches the sampled one, demonstrating both the precision of the theoretical analysis and the accuracy of the numerical methods. In addition, the use of high-performance frameworks enables the estimation to be carried out within reasonable times, without requiring many computational resources. Note that the yellow curve for the likelihood function exhibits a pronounced peak around 9:50 that appears much higher than the corresponding blue histogram bins (i.e., the empirical arrival time distribution). This is expected: the empirical arrival-time density is aggregated over discrete time intervals, whereas the yellow curve represents a likelihood function defined on a continuous domain. Because the continuous peak is much narrower than the histogram bins, its height is correspondingly larger.

### Performance of the MLE methods

Next, we investigate the performance of the method by performing a complete simulation-estimation cycle and plotting contour plots of slices of the likelihood function. Our primary interest lies in understanding how the parameters that maximize the likelihood deviate from the true value ***θ*** used to generate the dataset.

Due to the high dimensionality of the parameter space (five dimensions), direct visualization is infeasible. However, by fixing a subset of parameters—typically at their true values used in data generation—it is possible to examine two-dimensional slices of the likelihood function with respect to selected parameters. As the sample size increases, the minimum of these slices is expected to approach the global minimum of the full likelihood function.

**Figure 10:**
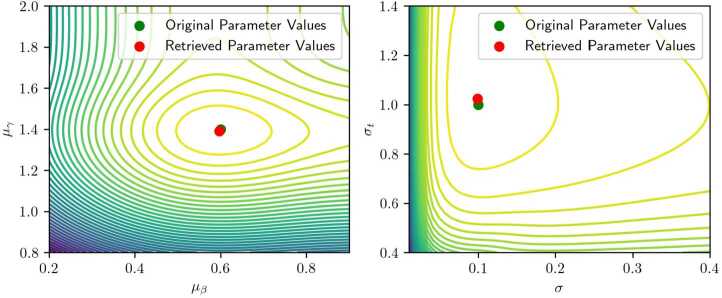
Contour plots representing convergence on the means $\mu _{\beta}$, $\mu _{\gamma}$ and on the variances *σ*, $\sigma _{t}$, with an average value of the variance *σ*. Parameters used for these plots are $\boldsymbol {\theta}= (0.6, 1.4, 9.5, 0.1, 1)$

Figure 10 illustrates the likelihood surface for a moderate value of $\sigma = 0.1$. The used parameters are $\boldsymbol {\theta}\triangleq (\mu _{\beta}, \mu _{\gamma}, \mu _{t}, \sigma, \sigma _{t})^{T} = (0.6, 1.4, 9.5, 0.3, 1)^{T}$, and the sample size of the population is $n = 1000$. The function is smooth and displays a well-defined minimum at the true parameter values, indicating favorable convergence properties even with a relatively small dataset. Also, for reliable computational convergence, it is ideal that the likelihood surface be sufficiently smooth and exhibit limited local minima, even if it is not convex.

From now on, we focus on the parameters $\mu _{\beta}$ and $\mu _{\gamma}$, which govern the mean of the scheduling preference distribution. These parameters yield the most interpretable behavior when visualized. Among the full parameter set, the variance parameter *σ* is observed to have the greatest influence on the shape of the likelihood landscape.

**Figure 11:**
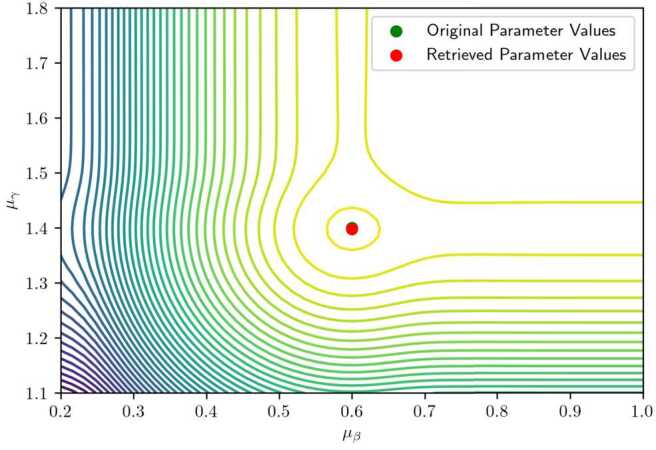
Contour plot of a two-dimensional slice of the likelihood function, with a higher value of the variance. The used parameters are here $\boldsymbol {\theta}= (0.6, 1.4, 9.5, 0.03, 1)$

When the standard variance *σ* is reduced to 0.03 (Fig. 11), the surface becomes relatively flat across a wide range of values for $\mu _{\beta}$ and $\mu _{\gamma}$, except in a narrow region near the true minimum. This behavior is consistent with the underlying data-generating process: for small variances, the observed arrival times concentrate near the extremes (early or late), and shifting the mean parameters has little effect on the likelihood unless the peak aligns precisely with the observed data. Consequently, large flat regions can emerge in the likelihood surface.

**Figure 12:**
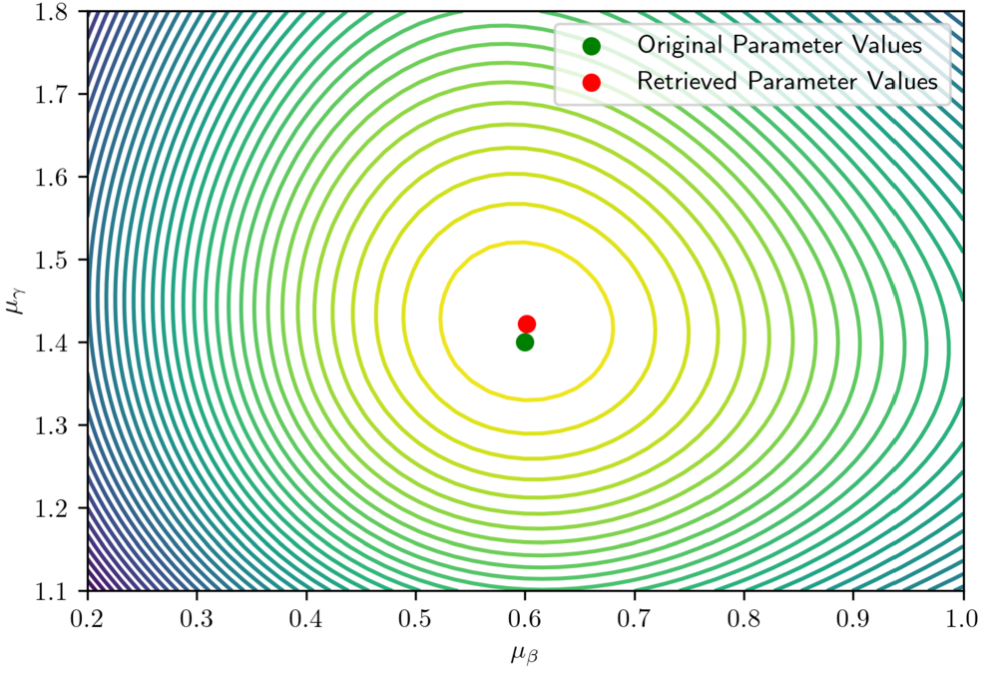
Contour plot of a two-dimensional slice of the likelihood function when the variance is set to $\sigma = 1 $

Conversely, with high variance $\sigma = 1$ (Fig. 12), the minimum becomes broader and shallower, potentially deviating from the true parameter values. This is attributable to increased dispersion in observed arrival times and diminished sensitivity of the likelihood function to changes in mean parameters. In such cases, a larger dataset is required to recover the true parameters with comparable accuracy.

We compare the obtained ***θ*** from MLE with the true value. For datasets of 1000 observations, the MLE method yields relative estimation errors typically below 5% for moderate and low variance settings ($\sigma = 0.3$ and 0.03), and up to 10% under high variance ($\sigma = 1$). These results are consistent across different parameter configurations. While the absolute accuracy of the estimates may appear modest, it is important to note the dimensionality of the problem and the presence of variance parameters, for which higher estimation error is often acceptable. The likelihood surfaces observed suggest that, with careful initialization and adequate data, reliable parameter recovery is feasible.

## Numerical simulation with field data

In this section, we test the effectiveness of our methods in scenarios from real-world traffic data. First, we describe in Sect. 6.1 the input data regarding the travel time profile function and distribution of the preference parameters, emphasizing the data processing for the PeMS data in Sect. 6.1.1. Then we discuss the generation of arrival times on different days with different congestion patterns in Sect. 6.2. Last, we examine the effectiveness of our method on different days in Sect. 6.3.

### Input data

#### Travel time profile function

In contrast to the assumed travel time profile function in Sect. 5.1.1, now we turn to travel time profiles in the real world by resorting to the California Performance Measurement System (PeMS) data. The dataset is collected from nearly 40,000 loop detectors in California, in which traffic flow and occupancy are collected at 30-second intervals, and later aggregated and presented on 5-minute intervals [19]. Specifically, data about the first six months of 2017 will be used in this study.

**Figure 13:**
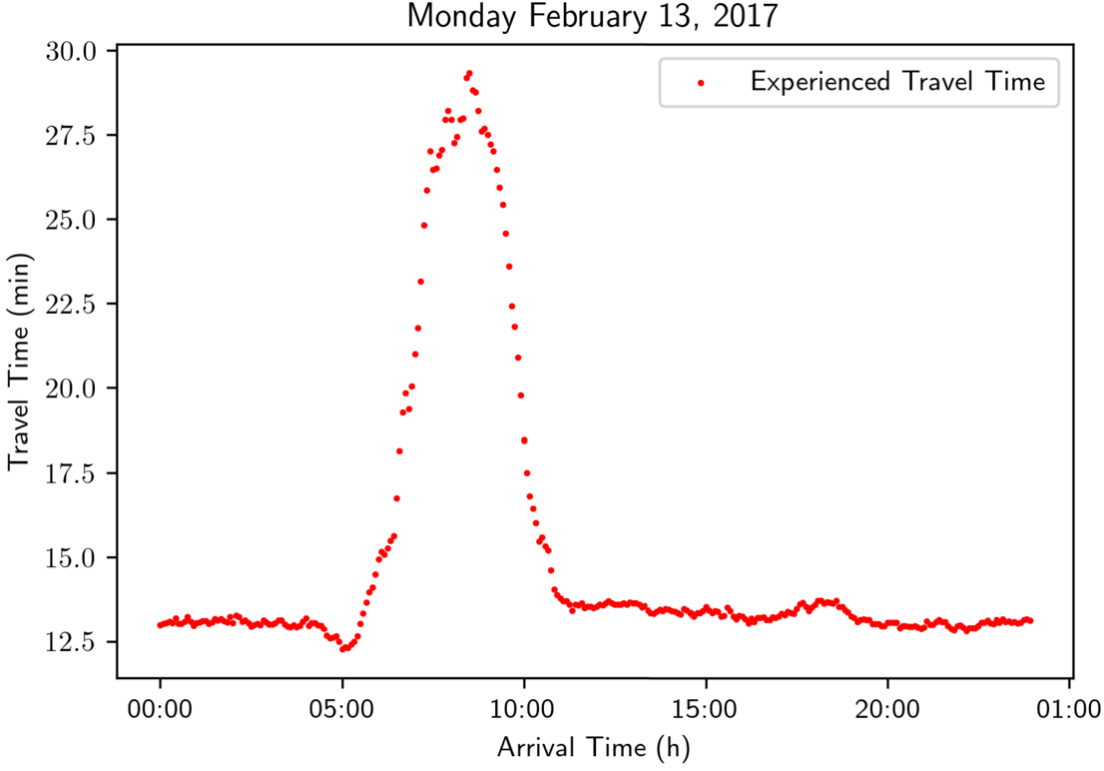
Experienced travel time on Highway 101 in the direction North on February 13, 2017, processed based on PeMS data

The original PeMS data captures the simultaneous travel time in road segments, but not the experienced travel time by travelers at different times of the day. To approximate the experienced travel time, we discretely integrate the reported speeds over different road segments [20]. As an example, Fig. 13 shows the processed data on the 13th of February, 2017.

Based on the approximated experienced travel time at discrete time intervals, we continue to generate a travel time profile function by fitting the discrete data points.^3^ In particular, we assume that the travel time profile function admits a format of the Skewed Super-Gaussian (SSG) function, i.e.,

26 $$ tt(t; \mu , \sigma , a, b, c) = \frac{e^{-|t-\mu |^{b}}}{\sigma} \frac{1}{1 + e^{-a(t - \mu )}} + c. $$

It is similar to the usual super-gaussian, to which we add a logistic multiplicative term to take into account the possible asymmetry of travel time data.^4^

**Figure 14:**
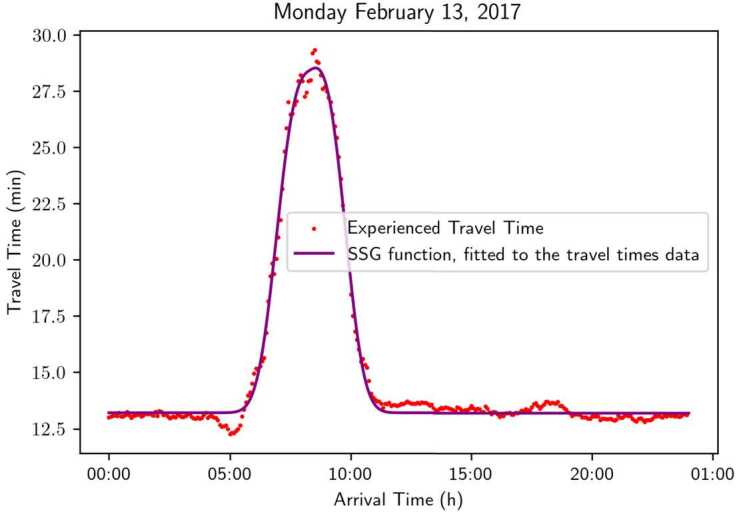
Experienced travel times data for the 13th of February, 2017, plotted alongside a fitted Skewed Super-Gaussian function

We use the Levenberg-Marquardt algorithm [21] to fit the data to the above-mentioned function. Figure 14 shows the fitted function on actual data. We can observe that the function precisely approximates the vast majority of the data points, while retaining the characteristics mentioned in Assumption 2. Namely, the function is concave in the middle while convex in both ends.

#### Distribution of the preference parameters

Again, we assume that *β*, *γ*, and $t^{*}$ are independent and they both conform to some Gaussian distribution, with *β* and *γ* sharing the same variance, as introduced in Sect. 5.1.2.

It is worth noting that the means of *β* and *γ* are carefully chosen in accordance with the shape of the real travel time profile. If the means of *β* and *γ* are extremely high (e.g., higher than $\beta _{max}$ and $\gamma _{max}$, respectively), then users are extremely reluctant to schedule displacement from their desired arrival time. The congestion would not actually affect their arrival times. In this case, it is therefore difficult to infer the actual preference since we can only observe their choices of being on time regardless of the congestion.

Thus, we focus on the case when the means of *β* and *γ* are intermediate, such that they are sufficiently smaller than $\beta _{max}$ and $\gamma _{max}$, respectively. Then we can observe sufficient early and late arrivals as input. Also, we notice that in practice, $\beta _{max}$ and $\gamma _{max}$ for many real travel time profiles can be relatively small [18]. This indicates that our method is more effective in estimating the preferences of populations with relatively low scheduling displacement penalty. We will discuss later in Sect. 7 about possible remedies for future work.

### Generating arrivals of multiple days

In this section, we consider the case when the input data contains the arrivals of a population across multiple days with different travel time profiles.

To do so, we still first sample a population with different *β*, *γ*, and $t^{*}$ from Gaussian distributions, as described in Sect. 5.2. Then we optimize the cost function for each individual in the population on each day, with the corresponding travel time profiles.

**Figure 15:**
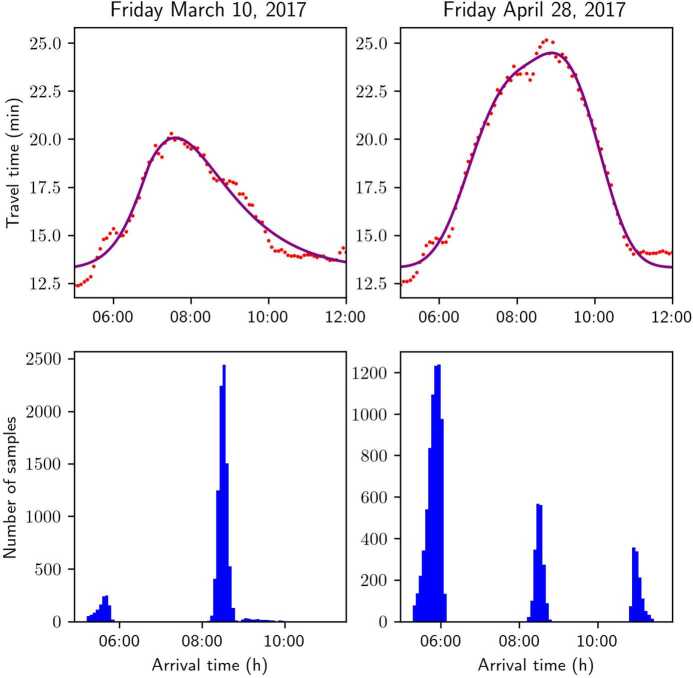
Travel time profiles and sampled arrivals on different days

Figure 15 shows the variations of sampled arrival times between two different days, with the population being fixed. In the top row, data points of real experienced travel time are shown in red, alongside the fitted function. In the bottom row, histograms of $n=10{,}000$ sampled arrival times for the two days are shown. Note that the parameters for sampling the synthetic datasets are the same: for both plots, $\boldsymbol {\theta}= (0.05, 0.08, 8.5, 0.02, 0.1)$. We notice that the sampled arrivals shown on the bottom row differ significantly: the wider peak of the travel time relative to April 28th implies that, for this day, on-time, early, and late arrivals coexist. On the other hand, a narrower peak entails closer CEA and CLA intervals and, as a consequence, having arrival time observations from different days contributes to the variety of choices and can potentially improve the performance of the estimation method.

**Figure 16:**
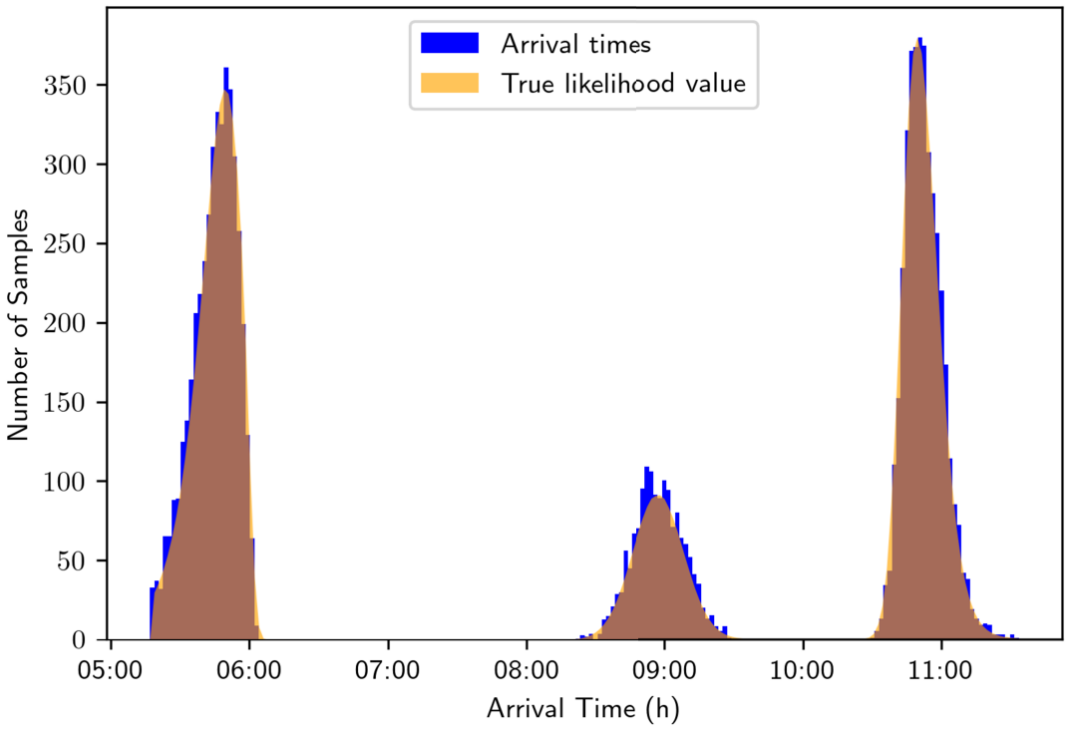
Empirical density of sampled arrival times, plotted with the value of the theoretical likelihood function. The travel time profile function is fitted to data relative to Friday, April 28th, and the parameters used for plotting are $\boldsymbol {\theta}= (0.05, 0.08, 9.0, 0.02, 0.2)$

Again, the histogram of sampled arrival times is plotted in Fig. 16 and compared with the theoretical likelihood. It shows that the theoretical density (that is, the value of the likelihood function) still closely follows the empirical distribution of the samples, which is consistent with the results from Sect. 5.2.2.

### Performance of the MLE methods

**Figure 17:**
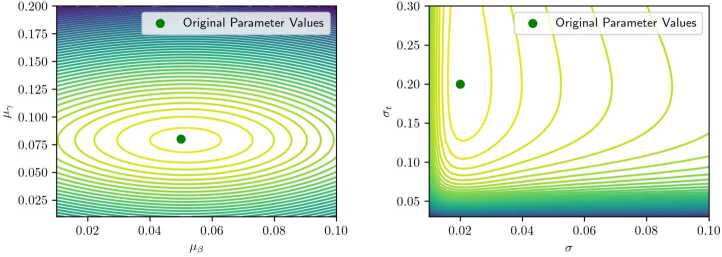
Contour plots that represent how the likelihood varies on two-dimensional slices of its domain. These plots refer to travel times data on Friday, April 28th, and the parameters used for plotting are $\boldsymbol {\theta}= (0.05, 0.08, 9.0, 0.02, 0.2)$

First, we demonstrate that the likelihood function is indeed minimized by the true parameters of the distribution of behavioral parameters. Given a dataset of arrival times, in Figure 17, we plot the likelihood under different combinations of $\mu _{\beta}$ and $\mu _{\gamma}$ in the left subfigure and $\delta _{t}$ and *δ* in the right subfigure, respectively. The rest of the parameters are fixed to their true value. We can observe that the true parameter lies in a minimum of the contour, and that the likelihood function is reasonably well behaved around it. This confirms the effectiveness of the MLE method to recover the true parameter.

**Figure 18:**
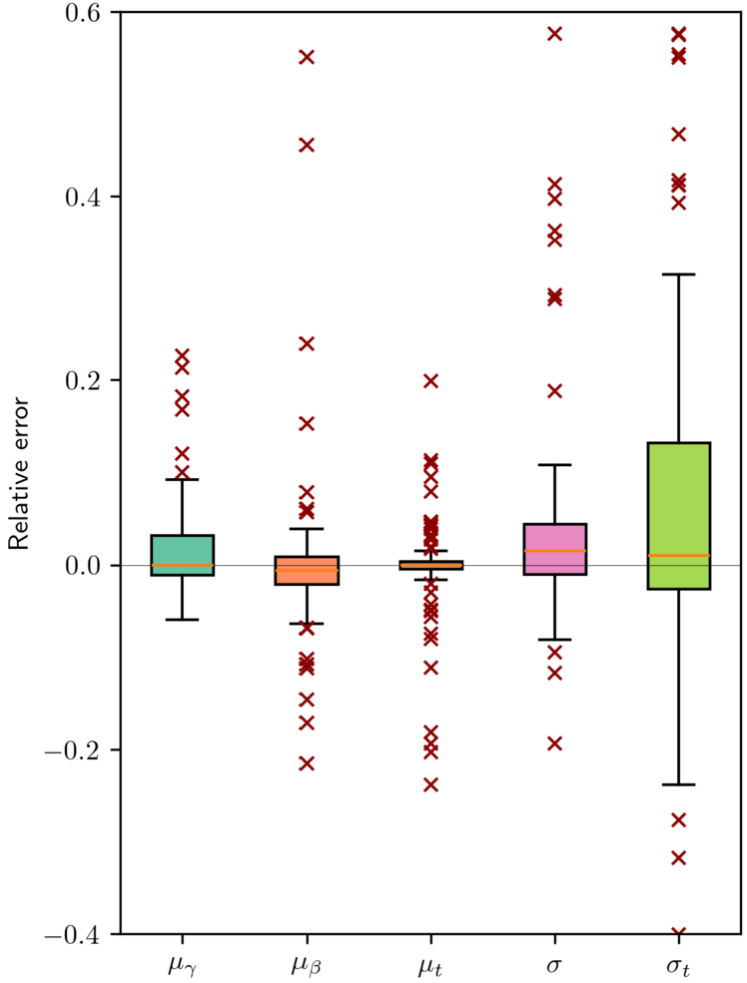
Boxplot of relative errors of 120 iterations of the evaluation framework of the developed method. The errors are computed by subtracting the result of the iteration from the original parameters, and normalizing the result by computing their ratios with typical values of the parameters

To further examine the performance of our method, we employ our estimation method on 120 different days randomly sampled from the first six months of 2017, and obtain the estimated distribution for the preference parameters for each day. Figure 18 shows the boxplot of the statistics regarding the gap between the estimated parameters and the true parameter. It shows that the method precisely estimates the means of all the parameters: the scheduling parameters and the desired arrival times. Meanwhile, a small bias (around 1% of error) is found in the estimation of the variances. We postulate that this is due to the structure of the problem: when the optimizer converges to a local minimum of the likelihood, the local minimum typically presents high values of the variance, since increasing the variance yields satisfactory results regardless of the values of the means $\mu _{\beta}$, $\mu _{\gamma}$, and $\mu _{t}$.

## Conclusion

This paper has proposed a structural framework to infer the distribution of commuters’ scheduling preferences using only RP data, thereby avoiding the limitations of SP surveys (see [22]). A key contribution lies in the detailed analysis of the generalized cost function, which remains non-trivial even under quasi-concave travel time profiles. This analysis establishes a geometric connection between early and late arrival intervals and the slope of the travel time function, offering both a behavioral interpretation of commuters’ scheduling choices and a tractable basis for estimation. Building on this, we derived a closed-form likelihood expression and developed an optimization method capable of recovering the global minimum of the likelihood. This allows the joint estimation of the distribution of schedule preference parameters and the distribution of desired arrival times.

The framework has been validated on synthetic datasets, where it accurately recovers population-level parameters, and further tested on empirical traffic data. Note that our method can be extended to infer which population groups exhibit a stronger tendency to arrive early. When multiple groups can be clearly distinguished from one another (e.g., by vehicle type or payment records), separate datasets of arrival times can be obtained for each group. This enables the application of our method to each group individually, allowing us to infer the distribution of scheduling preferences across different subpopulations.

While these results confirm its potential for real-world applications in travel behavior analysis and transportation planning [23], discrepancies remain—particularly in the estimation of desired arrival times. These can be partly explained by the flatter slopes of observed travel time profiles compared with those implied by the benchmark parameters reported in the literature. We also note that the travel-time profiles are relatively flat during non-rush hours, which further complicates the application of our method to more general cases.

To address this issue, several extensions have been identified. One option is to preserve the core theoretical structure while introducing richer forms of heterogeneity, interpreting literature-based parameters as population-level means within more flexible, possibly nonparametric, distributions. This line of work naturally lends itself to Bayesian approaches ([24]). A second extension relaxes the assumption of deterministic travel times by treating perceived travel times as stochastic, thereby capturing both day-to-day variability and perceptional noise. (For a discussion of the relationship between deterministic formulations and the stochastic-master equation-approach, see [25]). A third possibility involves incorporating unobserved heterogeneity (see [4]), which leads to probabilistic choice models whose first-order conditions generate more realistic slopes consistent with empirical evidence.

Finally, the proposed methodology is designed to be integrated into simulation platforms such as METROPOLIS,^5^ which require robust estimates of behavioral parameters for calibration (see, for example, [26]). Overall, this work provides a foundation for inferring scheduling preferences from large-scale RP data and opens new avenues for reconciling theoretical models with empirical observations.

## Figures and Tables

**Figure 19:**
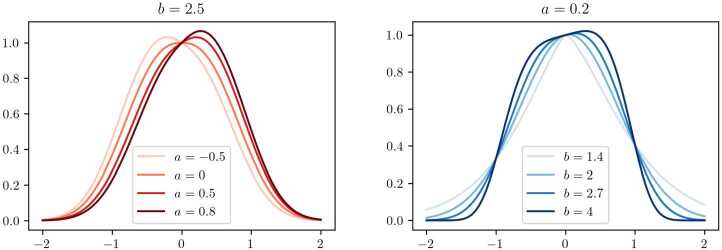
Skewed Super-Gaussian function, with varying shape parameters but constant values for *μ* and *σ*. The plots show how the different parameters act on the asymmetricity and flatness of the function. Notably, the subsequent convexity and concavity of the function are preserved

## Data Availability

No datasets were generated or analysed during the current study.

## Appendix A: Proof of Proposition 1

### A.1 Early and late minimal travel costs

To characterize the minima of the travel cost function, we first obtain the following lemmas.

#### Lemma 1

*Any two critical early arrival* (*CEA*) *intervals or two critical late arrival* (*CLA*) *intervals are disjoint*.

#### Proof

Suppose that there are two CEA intervals $[t_{i}^{e}, t_{f}^{e}]$ and $[t_{i}^{e'}, t_{f}^{l'}]$ overlapping with each other, i.e., $t_{i}^{e'} \le t_{f}^{l}$ and $t_{i}^{e} \le t_{f}^{l'}$. Then for any *t* in the overlapped part $[t_{i}^{e'}, t_{f}^{l}]$, the condition still holds:

A.1 $$ tt(t ) \ge tt(t_{i}^{e}) + \beta (t - t_{i}^{e} ). $$

Thus, the interval $[t_{i}^{e}, t_{f}^{l'}]$ is an EA interval and it strictly contains $[t_{i}^{e}, t_{f}^{l}]$ and $[t_{i}^{e'}, t_{f}^{l'}]$. This contradicts the definition of CEA intervals. The proof for CLA intervals is similar and therefore omitted here. □

Lemma 1 can be interpreted in this way: if two such intervals overlapped, they could be merged into a strictly larger interval that still satisfies the defining inequality. But “critical” intervals are, by definition, maximal ones not contained in others. Hence, overlaps cannot occur. Intuitively, CEA/CLA intervals are “time windows where it pays to shift arrivals”. Overlap would mean redundancy, so only disjoint maximal windows exist. By Lemma 1, each desired arrival time $t^{*}$ can fall into at most one CEA (CLA) interval, which leads to the following lemma.

#### Lemma 2

*Under Assumption* 1, *given any travel time profile* *tt*, *early arrival penalty* *β*, *and desired arrival time* $t^{*}$, *the minimal early arrival cost is*

A.2 $$ C_{e}^{\textit{opt}} = \textstyle\begin{cases} C(t_{i}^{e}), \textit{if }\exists [t_{i}^{e}, t_{f}^{e}] \in E \textit{ such that } t^{*} \in [t_{i}^{e}, t_{f}^{e}], \\ tt(t^{*}), \textit{otherwise}, \end{cases} $$

*where* *E* *denotes the set of all critical early arrival* (*CEA*) *intervals*.

#### Proof

Recall that the minimal early arrival cost $C_{e}^{opt}$ is defined as $C_{e}^{opt} = \min _{t \le t^{*}} C(t)$.

(1) We start with cases when there exists some CEA interval $[t_{i}^{e}, t_{f}^{e}]$ such that $t^{*} \in [t_{i}^{e}, t_{f}^{e}]$. To show that $C_{e}^{opt}$ is achieved at $t_{i}^{e}$, it is equivalent to show that $C(t) \ge C(t_{i}^{e}) $ for any $t \le t^{*}$. We proceed to show that $C(t) \ge C(t_{i}^{e})$ in two cases when $t \in [t_{i}^{e}, t^{*}]$ and $t < t_{i}^{e}$, respectively.

Case 1: $t \in [t_{i}^{e}, t^{*}]$. Recall that the trip cost at any $t \in [t_{i}^{e}, t^{*}]$ can be written as $C(t) = tt(t) + \beta (t^{*} - t)$. Using Inequality (2) from Definition 1 of CEA intervals, i.e., $tt(t ) \ge tt(t_{i}^{e}) + \beta (t - t_{i}^{e} )$, $\forall t \in [t_{i}^{e}, t_{f}^{e}]$, we obtain that $C(t) \ge tt(t_{i}^{e}) + \beta (t - t_{i}^{e} ) + \beta (t^{*} - t) = C(t_{i}^{e})$, $\forall t \in [t_{i}^{e}, t_{f}^{e}]$. Thus, we have $C(t) \ge C(t_{i}^{e}) $ for any $t \in [t_{i}^{e}, t^{*}]$.

Case 2: $t < t_{i}^{e}$. We prove that $C(t) \ge C(t_{i}^{e}) $ for any $t < t_{i}^{e}$ by contradiction. Suppose that there exists some $t'< t_{i}^{e}$ such that $C(t') < C(t_{i}^{e})$. Define a set

$$ T = \{t \in [t', t_{i}^{e}] \mid tt(t') + \beta (t - t' ) = tt(t )\}, $$

and let $t'' = \max (T)$. Apparently, $C(t) = C(t')$ for any $t \in T$. Next, we will prove that the interval $[t'', t_{f}^{e}]$ is a CEA interval, which contains $[t_{i}^{e}, t_{f}^{e}]$, contradicting the definition of a critical early arrival interval.

To do so, we prove that the following condition holds by Definition 1: $tt(t'') + \beta (t - t'' ) \le tt(t )$, $\forall t \in [t'', t_{f}^{e}]$.

First, we show that $tt(t'') + \beta (t - t'' ) \le tt(t )$ holds for any $t \in [t_{i}^{e}, t_{f}^{e}] $. Recall that $[t_{i}^{e}, t_{f}^{e}]$ is a CEA interval, and therefore by definition the following inequality holds: $tt(t_{i}^{e}) + \beta (t - t_{i}^{e} ) \le tt(t )$, $\forall t \in [t_{i}^{e}, t_{f}^{e}]$. Given that $C(t') < C(t_{i}^{e})$ by assumption and $C(t'') = C(t')$ by definition, we have $C(t'') < C(t_{i}^{e})$. Thus, we have $tt(t'') + \beta (t - t'' ) < tt(t_{i}^{e}) + \beta (t - t_{i}^{e} )$, $\forall t \in [t_{i}^{e}, t_{f}^{e}] $. That is, $tt(t'') + \beta (t - t'' ) < tt(t )$, $\forall t \in [t_{i}^{e}, t_{f}^{e}]$.

Second, we show that $tt(t'') + \beta (t - t'' ) \le tt(t )$ holds for any $t \in [t'', t_{i}^{e}]$. Let $f(t)= tt(t) + \beta (t'' - t ) - tt(t'' ) $, which is continuous. We have shown above that $tt(t'') + \beta (t_{i}^{e} - t'' ) < tt(t_{i}^{e})$, i.e., $f(t_{i}^{e}) <0$. Also, we have $f(t'') =0$ by definition.

Suppose that there exists some $t \in (t'', t_{i}^{e})$ such that $tt(t) + \beta (t'' - t ) > tt(t'' )$, i.e., $f(t) <0$. By the continuity of $f(t)$, there must be some $t''' \in (t, t_{i}^{e})$ such that $f(t''')=0$, i.e., $tt(t''') + \beta (t'' - t''' ) = tt(t'' )$. Therefore, we have $t''' \in T$ and $t''' > t''$, which contradicts the condition that $t'' =\max (T)$. Thus, we must have $tt(t'') + \beta (t - t'' ) \le tt(t )$ holds for any $t \in [t'', t_{i}^{e}]$.

(2) Now we turn to discuss the case when $t^{*}$ does not fall into any CEA intervals. Again, we prove by contradiction. Suppose that there exists some $t' < t^{*}$ such that $C(t') < C(t^{*})$. Redefine a set $T = \{t \in [t', t^{*}] \mid tt(t') + \beta (t - t' ) = tt(t )\} $, and still let $t'' = \max (T)$. Since $C(t') < C(t^{*})$, we have $tt(t') + \beta (t^{*} - t') < tt(t^{*})$. Using that $tt(t'') + \beta (t - t'' ) = tt(t') + \beta (t - t' ) = tt(tt') $, we have $tt(t'') + \beta (t^{*} - t'') < tt(t^{*})$. Also, the following equation naturally holds: $tt(t'') + \beta (t'' - t'') = tt(t'')$. By continuity and contradiction, we are able to show that $tt(t'') + \beta (t - t'') \le tt(t)$, $\forall t \in [t'', t^{*}]$. That is, $[t'', t^{*}] $ is an EA interval. And there always exists some CEA interval that contains $[t'', t^{*}]$. Therefore, $t^{*}$ would fall into some CEA interval, which contradicts. The proof is completed. □

Lemma 2 shows that the minimum of the travel cost function before $t^{*}$ is achieved at the left boundary of a CEA interval if it contains $t^{*}$. If $t^{*}$ is not inside any CEA interval, the best option is to arrive exactly at $t^{*}$. Intuitively, if arriving early is “worth it”, the very first point where this becomes beneficial is best. Otherwise, one should aim to be on time.

In parallel, we can prove the following lemma concerning late arrivals. The proof is similar to the proof for Lemma 2 and therefore omitted here.

#### Lemma 3

*Under Assumption* 1, *given any travel time profile* *tt*, *γ*, *and* $t^{*}$, *the minimal late arrival cost is*

A.3 $$ C_{l}^{\textit{opt}} = \textstyle\begin{cases} C(t_{f}^{l}), \textit{if }\exists [t_{i}^{l}, t_{f}^{l}] \in L \textit{ such that } t^{*} \in [t_{i}^{l}, t_{f}^{l}], \\ tt(t^{*}), \textit{otherwise,} \end{cases} $$

*where* *L* *denotes the set of all critical late arrival* (*CLA*) *intervals*.

Lemma 3 shows that the minimal late arrival cost after $t^{*}$ is either at the right endpoint of a CLA interval containing $t^{*}$, or at $t^{*}$ otherwise. By symmetry with Lemma 2, in CLA intervals, waiting longer reduces cost, so the best is the far-right point of the CLA interval. Otherwise, being on time dominates. Intuitively, arriving late only pays off if congestion is dropping quickly enough. Then, the best is to push the arrival as far as possible within that profitable zone.

### A.2 Characterization of the minima of travel cost

Lemmas 2 and 3 respectively reveal the early and late minimal travel costs $C_{e}^{\mathrm{opt}}$ and $C_{l}^{\mathrm{opt}}$ for any desired arrival time $t^{*}$. Then we can further identify the (globally) minimal travel cost $C^{\mathrm{opt}}$ by comparing $C_{e}^{\mathrm{opt}}$ and $C_{l}^{\mathrm{opt}}$. In particular, we proceed with the analysis based on whether the desired arrival time $t^{*}$ falls into a CEA interval or a CLA interval given *β* and *γ*.

(1) When both $\mathcal {E}(t^{*})$ and $\mathcal {L}(t^{*})$ are empty, we have $C_{e}^{opt} = tt(t^{*})$ by Lemma 2 and $C_{l}^{opt} = tt(t^{*})$ by Lemma 3. Therefore, the corresponding minimal travel cost is $C^{opt} = tt(t^{*})$. Namely, it is optimal to arrive on time.

(2) When $t^{*}$ falls into a CEA interval but not any CLA interval, we have $C_{e}^{opt} \le tt(t^{*})$ by Lemma 2 while $C_{l}^{opt} = tt(t^{*})$ by Lemma 3. Then we can immediately conclude that $C_{e}^{opt} \le C_{l}^{opt}$ and therefore the corresponding minimal travel cost is $C^{opt} = C_{e}^{opt}$.

(3) Similarly, we can prove that the minimal travel cost is $C^{opt} = C_{e}^{opt}$ when $t^{*}$ falls into a CLA interval but not any CEA interval.

## Appendix B: Proof of Proposition 2

Since $[t_{i}^{e}, t_{f}^{e}]$ and $[t_{i}^{l}, t_{f}^{l}]$ are respectively a CEA interval and a CLA interval, by Propositions 2 and 3, the mimimal early and late arrival costs, $C_{e}^{opt}$ and $C_{l}^{opt}$ are achieved at $t_{i}^{e}$ and $t_{f}^{l}$, respectively, i.e, $C_{e}^{opt} = C(t_{i}^{e}) = tt(t_{i}^{e}) + \beta (t^{*} - t_{i}^{e})$, and $C_{l}^{opt} = C(t_{f}^{l}) = tt(t_{f}^{l}) + \gamma ( t_{f}^{l}-t^{*})$.

Note that by the expression of the desired arrival time threshold $\bar{t}^{*}$ in Equation (5), we have $tt(t_{i}^{e}) + \beta (\bar{t}^{*} - t_{i}^{e}) = tt(t_{f}^{l}) + \gamma (t_{f}^{l}- \bar{t}^{*})$. Since $C_{e}^{opt} = tt(t_{i}^{e}) + \beta (t^{*} - t_{i}^{e})$, and $C_{l}^{opt} = tt(t_{f}^{l}) + \gamma ( t_{f}^{l}-t^{*})$, we have $C_{e}^{opt} = C_{l}^{opt} $ when $t^{*} = \bar{t}^{*}$.

Furthermore, it is straightforward to see that $\partial C_{e}^{opt}/\partial t^{*} >0$ and $\partial C_{l}^{opt}/\partial t^{*} <0$, which leads to that $\partial (C_{e}^{opt} - C_{l}^{opt}) /\partial t^{*} >0 $. Thus, we have $C_{e}^{opt} > C_{l}^{opt}$ when $t^{*} > \bar{t}^{*}$, and $C_{e}^{opt} < C_{l}^{opt}$ when $t^{*} < \bar{t}^{*}$. The proof is completed.

## Appendix C: Proof of Proposition 3

Given any *β*, by the definition of CEA intervals in inequality (2) we have

$$ tt_{a}(t) \geq tt_{a}(t_{e}^{i}(\beta )) + \beta [t - t_{i}^{e}( \beta )], \forall t \in [t_{i}^{e}(\beta ), t_{f}^{e}(\beta )]. $$

Consider some $\beta ' < \beta $. Then we have $\beta '[t - t_{i}^{e}(\beta )] < \beta [t - t_{i}^{e}(\beta )]$, and therefore

$$ tt_{a}(t) \geq tt_{a}(t_{e}^{i}(\beta )) + \beta '[t - t_{i}^{e}( \beta )], \forall t \in [t_{i}^{e}(\beta ), t_{f}^{e}(\beta )]. $$

Thus, $[t_{i}^{e}(\beta ), t_{f}^{e}(\beta )]$ is a EA interval under $\beta '$, which is always contained by a CEA $[t_{i}^{e}(\beta '), t_{f}^{e}(\beta ')] $, i.e., $t_{i}^{e}(\beta ') \le t_{i}^{e}(\beta )$ and $t_{f}^{e}(\beta ') \ge t_{f}^{e}(\beta )$. The discussion regarding CLA intervals is similar and therefore omitted. The proof is completed.

## Appendix D: Proof of Proposition 4

By Lemma 2, the left endpoint of a CEA interval is a minimizer of the cost function, i.e., $C(t_{i}^{e}) = \min _{t \le t^{*}} C(t)$ with $C(t) = tt(t) + \beta (t^{*} - t)$. Under Assumption 2, the function *tt* is twice differentiable. Thus, by the optimality condition, we have $tt'(t_{i}^{e}) = \beta $, and $tt''(t_{i}^{e}) \geq 0 $.

By Assumption 2, when $t < k_{1}$, $tt(t)$ is convex and increasing in *t*. Then $tt'(t)$ is monotonically increasing in *t* when $t< k_{1}$. Thus, there is at most one $t < k_{1}$ such that $tt'(t) = \beta $. When $t \in [k_{1}, k_{2}]$, $tt(t)$ is concave and therefore $tt''(t) \le 0$. When $t >k_{2}$, $tt(t)$ is concave and decreasing in *t*, i.e., $tt'(t)\le 0$. Therefore, there is at most one $t \in \mathcal{T}$ such that $tt'(t) = \beta $ and $tt''(t) \geq 0$. Namely, there is at most one CEA interval. Similarly, we can show that there is at most one CLA interval, which is omitted here. The proof is completed.

## Appendix E: Computational techniques for integrals

We begin by precalculating the value of $\beta _{max}$, $\gamma _{max}$, which depend solely on the travel time profile function *tt*. Under assumption 2, *tt* is twice differentiable. The coefficients are thus computed by running a gradient descent optimizer on the derivative of the travel time function.

Next, we explain how to compute the upper limits $\check{t}_{f}^{e}(\beta , \gamma )$ and $\check{t}_{i}^{l}(\beta , \gamma )$ of the integrals in Equations (17) and (23), respectively, and the lower limits $\beta _{0}(t)$ and $\gamma _{0}(t)$ of the integral in Equation (13).

### E.1 Upper limit

Given any $\beta \le \beta _{max}$ and $\gamma \le \gamma _{max}$, we need to update the value of $\check{t}_{f}^{e}(\beta , \gamma )$ and $\check{t}_{i}^{l}(\beta , \gamma )$ in (24). Recall that $\check{t}_{f}^{e}(\beta , \gamma )$ and $\check{t}_{i}^{l}(\beta , \gamma )$ are defined in a convoluted way: to compute $\check{t}_{f}^{e}(\beta , \gamma )$ and $\check{t}_{i}^{l}(\beta , \gamma )$, we need to obtain $\bar{t}^{*}$, $t_{f}^{e}$ and $t_{i}^{l}$ first, as described in Corollary 2.

First, as defined in Equation (5), the term $\bar{t}^{*}$ explicitly depends on $t_{i}^{e}$ and $t_{f}^{l}$. By Proposition 4, we have $tt'(t_{i}^{e})= \beta $ and $tt'(t_{f}^{l})= \gamma $. Equivalently, $t_{i}^{e} = \operatorname*{argmin}_{t} (tt(t) - \beta t)$ and $t_{f}^{l} = \operatorname*{argmin}_{t} (tt(t) + \gamma t)$. Thus, $t_{i}^{e}$ and $t_{f}^{l}$ can be found by running a simple gradient descent optimizer on the functions $tt(t) - \beta t$ and $tt(t) + \gamma t$.

Second, by Definitions 1, the endpoints $t_{f}^{e}$ and $t_{i}^{l}$ on the other sides depend on $t_{i}^{e}$ and $t_{f}^{l}$ as follows: $tt(t_{f}^{e}) = tt(t_{i}^{e}) + \beta (t_{f}^{e} - t_{i}^{e})$, $tt(t_{i}^{l}) = tt(t_{f}^{e}) + \gamma (t_{f}^{l} - t_{i}^{l}) $. Therefore, given $t_{i}^{e}$ and $t_{f}^{l}$, we can solve $t_{f}^{e}$ and $t_{i}^{l}$ using the bisection method. Then we are ready to obtain $\check{t}_{f}^{e}(\beta , \gamma )$ and $\check{t}_{i}^{l}(\beta , \gamma )$ by letting $\check{t}_{f}^{e}(\beta , \gamma ) = \min \{t_{f}^{e}, \bar{t}^{*}\}$, and $\check{t}_{i}^{l}(\beta , \gamma )= \max \{t_{i}^{l}, \bar{t}^{*}\} $.

### E.2 Lower limit

Given any $t \in \mathcal {T}$, we need to update the value of $\beta _{0}(t)$ and $\gamma _{0}(t)$ in (24). Recall that, by Corollary 1, there exists a threshold $\beta _{0}(t^{*})$ of *β* such that $t^{*} \notin \tilde{E}(\beta , \gamma )$ when $\beta > \beta _{0}(t^{*})$, and a threshold $\gamma _{0}(t^{*})$ such that $t^{*} \notin \tilde{L}(\beta , \gamma )$ when $\gamma > \gamma _{0}(t^{*})$.

To find such thresholds, we employ a bisection algorithm: progressively increasing the value of *β*, *γ* until $t^{*}$ is no longer in a CEA (respectively, CLA) interval, we find meaningful initial conditions. Note that by bisection, we are then able to estimate the coefficients with arbitrary precision.

Using the above-mentioned computation techniques, we perform numerical experiments in the following sections to demonstrate the effectiveness of our methods.

## Appendix F: The flexibility of the skewed super-Gaussian function

Figure 19 displays the flexibility of the obtained function: By varying the parameter *a*, the skewness of the function can be modified. On the other hand, changing the parameter *b* allows for increasing or decreasing the flatness of the function peak.

## Footnotes

1 When $tt'(t) < 1$ for all $t \in \mathcal{T}$, there is a one-to-one correspondence between any arrival time *t* and the departure time $t_{d} = t - tt(t)$ because $\partial t_{d}/\partial t > 0$ in this case. Then the arrival time choice problem is equivalent to the departure time choice problem in [6].

2 The resulting code is available at github.com/Piripuz/Reverse_ADL_Vickrey.

3 Alternatively, we can obtain a continuous travel time profile function through interpolation. However, the specification of function types allows us to constrain the function to some particular shapes, such as the bounds on convexity specified in assumption 2, which tends to be robust with outliers and noisy data.

4 An example of such flexibility is provided in Appendix F.

5 The METROPOLIS 2 model is available on the following website https://metropolis2.org/

## Abbreviations

OD: Origin–Destination
RP: Revealed Preference
SP: Stated Preference
TDM: Travel Demand Management
MLE: Maximum Likelihood Estimation
PDF: Probability Density Function
CEA: Critical Early Arrival
CLA: Critical Late Arrival
EA: Early Arrival
LA: Late Arrival
PeMS: California Performance Measurement System
SSG: Skewed Super-Gaussian
